# Differential *actinodin1* regulation in embryonic development and adult fin regeneration in *Danio rerio*

**DOI:** 10.1371/journal.pone.0216370

**Published:** 2019-05-02

**Authors:** Hue-Eileen Phan, Marissa Northorp, Robert L. Lalonde, Dung Ngo, Marie-Andrée Akimenko

**Affiliations:** Department of Biology, University of Ottawa, Ottawa, Ontario, Canada; Laboratoire de Biologie du Développement de Villefranche-sur-Mer, FRANCE

## Abstract

Actinotrichia are the first exoskeletal elements formed during zebrafish fin development. These rigid fibrils serve as skeletal support for the fin fold and as substrates for mesenchymal cell migration. In the adult intact fins, actinotrichia are restricted to the distal domain of the fin. Following fin amputation, actinotrichia also reform during regeneration. The *actinodin* gene family codes for structural proteins of actinotrichia. We have previously identified *cis-*acting regulatory elements in a 2kb genomic region upstream of the first exon of *actinodin1*, termed 2P, required for tissue-specific expression in the fin fold ectoderm and mesenchyme during embryonic development. Indeed, 2P contains an ectodermal enhancer in a 150bp region named *epi*. Deletion of *epi* from 2P results in loss of ectodermal-specific activity. In the present study, we sought to further characterize the activity of these regulatory sequences throughout fin development and during adult fin regeneration. Using a reporter transgenic approach, we show that a site within the *epi* region, termed *epi3*, contains an early mesenchymal-specific repressor. We also show that the larval fin fold ectodermal enhancer within *epi3* remains functional in the basal epithelial layer during fin regeneration. We show that the first non-coding exon and first intron of *actinodin1* contains a transcriptional enhancer and an alternative promoter that are necessary for the persistence of reporter expression reminiscent of *actinodin1* expression during adulthood. Altogether, we have identified *cis*-acting regulatory elements that are required for tissue-specific expression as well as full recapitulation of *actinodin1* expression during adulthood. Furthermore, the characterization of these elements provides us with useful molecular tools for the enhancement of transgene expression in adulthood.

## Introduction

The zebrafish fins, like all teleost fins, are supported by two types of exoskeletal elements: lepidotrichia and actinotrichia. The lepidotrichia are calcified, dermal bones that extend along the entire length of the ray. The actinotrichia are unmineralized collagenous, rigid fibrils that form brush-like bundles at the distal tips of the rays [1–3]. The actinotrichia are the first exoskeletal elements to form in the embryonic fins. There are two types of embryonic fins: the paired pectoral fins and the median fin. The former will give rise to the pectoral fins; the latter, to the unpaired fins (dorsal, caudal and anal fins). The fin fold of these embryonic fins consists of two sheets of ectodermal cells separated by fibres of actinotrichia [3–7], which are believed to maintain the structure of the early fin fold before the emergence of the lepidotrichia during fin outgrowth [8, 9]. Actinotrichia also serve as a scaffold for the distal migration of mesenchymal cells invading the fin fold [4, 5, 6, 9, 10].

As fins develop, lepidotrichia are forming. The actinotrichia persist but, become restricted to the distal end of the fin rays. Following amputation, the actinotrichia are also the first exoskeletal elements to form during the regeneration process, before the lepidotrichia. Actinotrichia occur in thick longitudinal bundles in the distal regions of the regenerate. The distal ends of the actinotrichia occupy the subepidermal space while the proximal ends are located in the mesenchymal compartment deep to the osteoblasts [3, 11]. As regeneration progresses towards the later stages, actinotrichia turn over occurs through synthesis and degradation at the distal and proximal ends, respectively [12]. As a result, the actinotrichia remain restricted to the distal tips of the lepidotrichia as the regenerate reaches its final length. Aside from providing mechanical support at the distal-most tissues of the fin regenerate, it has been proposed that the actinotrichia may also act as a substrate for the migration of mesenchymal cells that emerge from the blastema [11].

Actinotrichia are composed of elastoidin, which consist of collagenous and non-collagenous proteins that confer a combination of rigidity and flexibility to the fin fold [3, 6, 12]. The collagenous components are made up of type I and type II collagens [3]. The non-collagenous components are known as the actinodin proteins, which are tyrosine-rich proteins encoded by the *actinodin* (*and*) gene family [13]. Zebrafish possess four *actinodin-*encoding genes, *actinodin1-4* (*and1-4*). During embryonic development, the expression *of and1* and *and2* spatially and temporally correlate with actinotrichia formation [13]; their expression occurs, first, in the ectoderm as the fin fold is forming, then in the mesenchymal cells that are invading the fin fold [14]. During adult fin regeneration, *and1* is expressed in a subset of the basal epithelial layer in the distal region of the fin regenerate and in a subset of mesenchymal cells located deep to the osteoblasts within the regenerating fin rays [3, 11, 13].

*Cis*-acting regulatory elements of *and1* that are active during embryonic and early larval fin development were characterized using several *and1* zebrafish transgenic reporter lines [14]. The *2P* region spans a 1941bp region located immediately upstream of the first non-coding exon (the first bp of the first exon is denoted +1) of *and1* and contains a promoter as well as important regulatory elements that drive reporter expression that recapitulates *and1* expression in the fin fold. In *Tg(2Pand1*:*eGFP)* transgenic lines, the 2P region drives reporter expression within the migrating mesenchymal cells and ectodermal cells of the pectoral and median fin fold (PFF and MFF) [14]. Within the *2P* region is a 150bp fragment (positions -1117 and -975), termed *epi*. When *epi* is combined with a minimal promoter (the human-*beta-globin* promoter) in *Tg(epi*.*and1-βG*:*eGFP)*, reporter expression is observed within the ectoderm of the median and pectoral fin folds. The removal of *epi* or *epi3*, (a 22bp sequence within *epi*), from *2P* in *Tg(2PΔepi*.*and1*:*eGFP)* or *Tg(2PΔepi3*.*and1*:*eGFP)* transgenic lines, respectively, results in an absence of reporter expression in the overlying ectoderm, leaving only mesenchymal reporter expression [14].

To date, it has been shown that pharmacologic inhibition of Sonic Hedgehog signaling, Bone Morphogenetic Protein receptors and of Histone deacetylase 1 disrupts actinotrichia formation during the regenerative outgrowth phase following adult fin amputation [15–18]. It has also been shown that pulse-inhibition of either TGFβ/Activin-βA or FGF signaling results in the disruption of Actinodin deposition during regeneration. However, whether or not TGFβ/Activin-βA or FGF signaling directly regulates *and1* expression remains uncertain [11]. In the present study, we sought to characterize some of the key regulatory elements that govern the expression of the *actinodin1* gene (*and1*) during fin regeneration. We further extended the analysis of the activity of the *cis*-acting regulatory elements necessary for *and1* expression in the embryonic and early larval fin development to their role in adult fin regeneration. We characterized additional regulatory elements that are essential for controlling actinotrichia formation during adulthood and fin regeneration. In addition, we provide evidence for a repressor and an enhancer, within epi, that control the dynamics of the actinotrichia in specific regions of the regenerate. Altogether, we have generated a transgenic line that fully depicts endogenous and1 expression from the embryonic stage to adulthood.

## Materials and methods

### Zebrafish husbandry

All fish used in the experiments were maintained at 28°C with a photoperiod of 14 hours of light and 10 hours of darkness. Fish were fed regularly [19]. Animal care and experiments were performed in accordance with the standards of the Canadian Council on Animal Care and the regulations of the Ontario Animals for Research Act. The protocols (BL-271 and BL-245) were approved by the University of Ottawa Animal Care Committee.

### Fin amputations

Zebrafish were anesthetized by immersion in system water containing 0.17mg/ml tricaine [19]. Caudal fins were amputated two segments proximal from the first branch point of the lepidotrichia; referred to as standard cut. Fish were then returned to fresh system water to recover.

### Live imaging

Adult fish were anesthetized and placed on a 1% agarose plate with the caudal fins spread out. Zebrafish embryos, larvae and juveniles were anesthetized in E3 embryo medium containing 0.1 mg/ml tricaine. The plate was placed under a Leica MZ FLIII dissection microscope and images were taken using the AxioCam HSM digital camera and AxioVision AC software (Carl Zeiss). For live confocal imaging, fish were anesthetized and immersed in 0.17mg/ml tricaine in a petri dish. The caudal fins were flattened to the bottom of the petri dish with a slide hold-down (Warner Instruments 64–0248) and imaged with a water-immersion objective equipped on Nikon A1RsiMP Confocal. All images were processed using ImageJ (NIH).

### *In situ* hybridization

*In situ* hybridization (ISH) on longitudinal and transverse cryosections of at least 3 adult fin regenerates per probe were performed as previously by Smith et al. [20] with modifications described in McMillan et al. [21].

### Double fluorescence *in situ* hybridizations (FISH) on sections

Double FISH on longitudinal and transverse cryosections was adapted from protocols that were previously described by Welton et al. [22]. Fully adapted protocol is described by McMillan et al. [21].

Digoxigenin-labeled *and1* antisense RNA probes were generated using *and1* cDNA (2383 base pairs (bp) [13]. Dinitrophenol-labeled *eGFP* antisense RNA probes were generated using *eGFP* cDNA [23].

### Immunohistochemistry

Immunohistochemistry on longitudinal and transverse cryosections was adapted as previously described by Smith et al. [20]. Zns5 immunohistochemistry was adapted from a protocol that was previously described by Smith et al. [20]. Longitudinal cryosections of 4dpa fin regenerates were incubated with Zns5 (ZFIN), rabbit anti-And1/2 protein (Life Technologies) [13], or rabbit anti-green fluorescent protein (Life Technologies) antibodies at 1:200. Fluorescently labeled secondary antibodies Alexa Fluor 594 goat anti-mouse IgG (H+L) or Alexa Fluor 488 goat and anti-rabbit IgG (Invitrogen, A11001) were used at 1:500. Slides were counterstained with DAPI and mounted.

### Plasmid construction

The cloning and subcloning of the *(1117–1)EIand1*:*eGFP* region were performed following the standard cloning procedures of Sambrook & Russell [24]. The genomic sequence was amplified using the Epi forward primer (5’-GCTAGCTTTCGGAAACCCCAGAC-3’) and the intron reverse primer (5’-GGCGGATCCCTTGGATGAAATTAA-3’), and cloned in a pDrive cloning vector (Qiagen). The *1117–1+EI* region, consisting of 4992 bp in total, was then subsequently subcloned into a modified *pEGFP-N1* cloning vector via NheI and BamHI restriction sites. The CMV regulatory region was removed from the original pEGFP-N1 cloning vector and replaced by a Tol2 (left) arm between the AseI and NheI restriction sites and another Tol2 (right) arm was inserted at the AflII restriction site.

The 1941–1117 fragment from the 2P region and the region continuous from the 3’ end of *epi* to the end of the first intron (positions -1117 to +638) were sequentially subcloned into the *pEGFP-N1 tol2* cloning vector to generate the *2PΔepi-EIand1*:*eGFP* construct. These fragments were cloned into the NheI, KpnI and AgeI restriction sites of *pEGFP-N1 tol2*. The 1941–1117 fragment was amplified using the NheI-1941-2Pand1 FW (5’-GCTAGCGGTGAATTACAGCTTTAAGAC-3’) and 1117-Epi-and1-KpnI Rev (5’-GAGCTCAAATGTGGAAACATCTGGAAA-3’) primers. The region continuous from the 3’ end of *epi* to the end of the first intron was amplified using the KpnI-967-(After) Epi-and1 FW (5’-GAGCTCCGTTAACATAAAGCACAGATG-3’) and (+)-681-intron-and1-AgeI Rev (5’-GTCGACCTTGGATGAAATTAATTACAGCTT-3’) primers. Each of these fragments were amplified from the *2P-EIand1*:*eGFP* construct from Lalonde et al. [14].

Cloning of the *arCshha-EIand1*:*eGFP*construct: The first exon and intron of *and1* (termed *EI)* were amplified using the FW Exon1 (5’-AACAGTGGTGCAGTCGGG-3’) and Rev Intron1 (5’-GGCGGATCCCTTGGATGAAATT AA-3’) and cloned into pDrive. *EI* was digested with the EcoRI restriction enzyme and subcloned into the *pEGFP-N1 tol2* cloning vector. The *shha arC* fragment was digested from the *shh arC+200–1* construct from Lalonde et al. [14] using the NheI and EcoRI restriction enzymes and was subcloned into the *EIand1*:*eGFP* construct.

### Microinjections

Reporter constructs (final concentration of 100ng/mL) are co-injected with transposase RNA (final concentration of 50ng/mL) mixed with distilled water and 0.5% phenol red in one cell-stage zebrafish embryos.

### Additional transgenic lines

The *Tg(2Pand1*:*eGFP)* (five lines for this construct have been generated), *Tg(2P-EIand1*:*eGFP)* (two lines), *Tg(epi*.*and1-βG*:*eGFP)* (two lines), *Tg(2PΔepi*.*and1*:*eGFP)* (two lines) and *Tg(2PΔepi3*.*and1*:*eGFP)* (one line) transgenic lines were obtained from Lalonde et al. [14]. ~25 fish per line were analyzed and showed the same expression patterns. Please note the following construct/transgenic line name changes from Lalonde et al. [14]: *Tg(epi*.*and1-βG*:*eGFP)* from *Tg(epi+βG*:*eGFP)*, *Tg(2PΔepi*.*and1*:*eGFP)* from *Tg(2PΔepi*:*eGFP)*, *Tg(2PΔepi3*.*and1*:*eGFP)* from *Tg(2PΔepi3*:*eGFP)* and *Tg(2P-EIand1*:*eGFP)* from *Tg(2P+I*:*eGFP)*.

## Results

### Differential regulation of *and1* throughout development and adulthood

A time course analysis of GFP expression starting at 2 days post fertilization (dpf) and ending at 90dpf was performed on several *and1* reporter transgenic lines described in Lalonde et al. [14] to characterize the activity of the regulatory elements throughout fin development. The specific starting and ending time points of the time course were chosen according to the stages at which zebrafish are deemed as larvae and as sexually mature adults, respectively [25]. In this study, we focused on the posterior-most part of the MFF that gives rise to the caudal fin.

**Fig 1:**
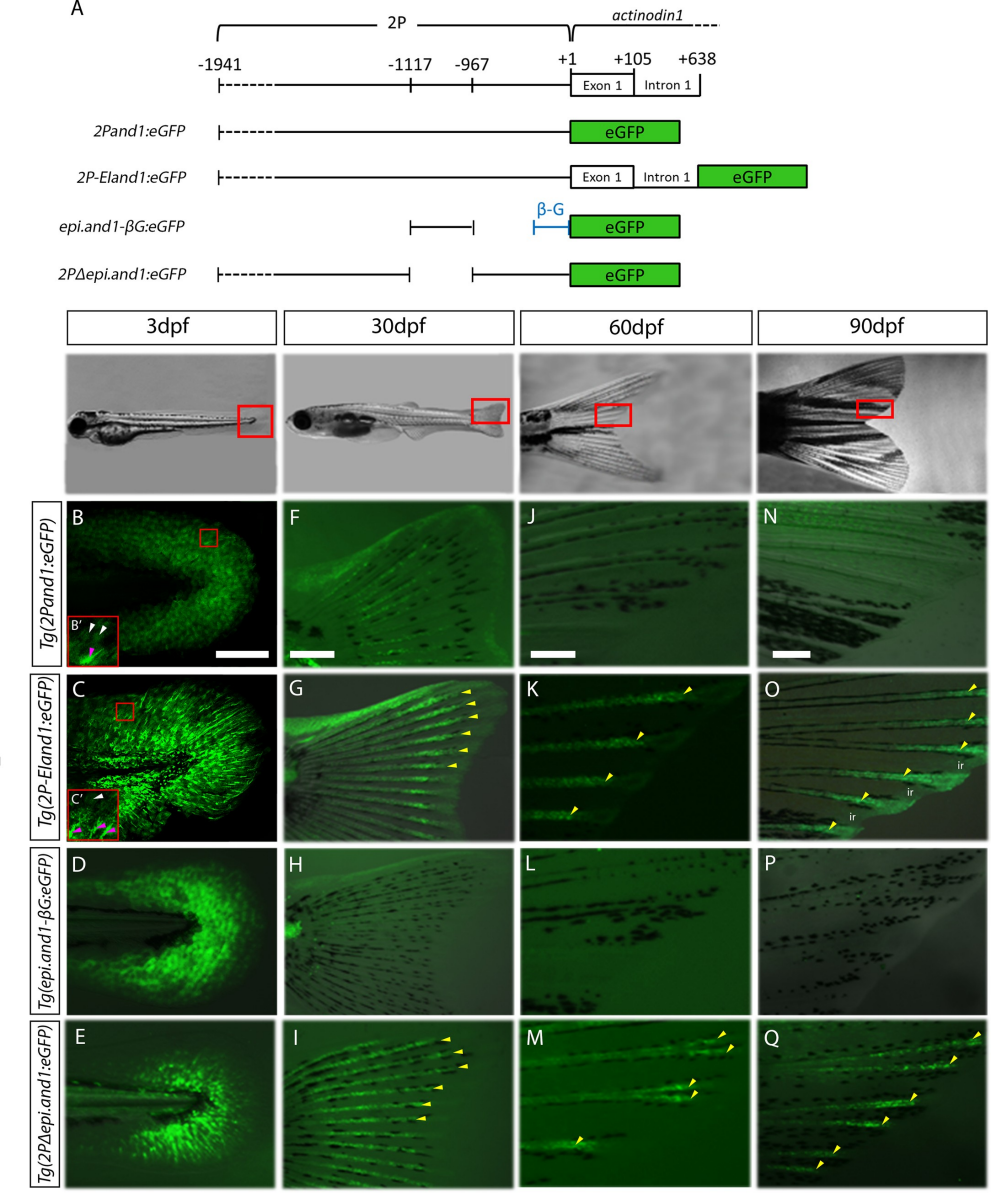
Differential regulation of *and1* during larval development and adulthood. (A) Schematic representation of transgenic reporter constructs. GFP expression of four *and1* reporter lines at 3dpf (B-E), 30dpf (F-I), 60dpf (J-M) and 90dpf (N-Q) showing fluorescent images merged onto bright field images. (B, B’, C, C’) confocal images (note: autofluorescence in the trunk tissue in C). Note: obtaining exactly the same plane of focus in the two transgenic lines is difficult; however, these images are simply to represent both transgenic lines have eGFP-positive ectodermal and mesenchymal cells. (B’, C’) 2x higher magnification on region boxed in red in panels B and C, respectively, showing the more elongated mesenchymal cells (pink arrowheads) in contrast to the hexagonally-shaped ectodermal cells (white arrowheads). (B, F, J, N) *Tg(2Pand1*:*eGFP)* (n = 5) expression in the ectoderm and fin ray mesenchyme fades after 30dpf. (C, G, K, O) *Tg(2P-EIand1*:*eGFP)* (n = 2) expression in the ectoderm and fin ray mesenchyme persists after 30dpf. (D, H, L, P) *Tg(epi*.*and1-βG*:*eGFP)* (n = 2) expression in the ectoderm is absent at 30dpf and onward; and (E, I, M, Q) *Tg(2PΔepi*.*and1*:*eGFP)* (n = 2) expression in the fin ray mesenchyme persists. Yellow arrowheads indicate fin ray-specific reporter expression. n = # of lines, ~25 fish/line. Scale bars = 200μm.

We previously showed that in the *Tg(2Pand1*:*eGFP)* transgenic line, the *2Pand1* regulatory elements and promoter (located at positions -1941 to +1) (Fig 1A) drives eGFP expression within the fin fold ectoderm and mesenchymal tissue of the developing larvae (Fig 1B) [14]. However, as will be further described below, reporter expression progressively disappears in *Tg(2Pand1*:*eGFP)* transgenic larvae and adult fish (Fig 1F, 1J and 1N). Therefore, a new transgenic line, containing additional genomic sequence was made in the aim to recapitulate *and1* endogenous expression observed in the larval and adult fins. In this new transgenic line, *Tg(2P-EIand1*:*eGFP)*, the transgene includes, in addition to the *2Pand1* fragment, the first exon (non coding exon) and intron of *and1*, which are collectively referred to as the *EI* region (located at positions +1 to +638) (Fig 1A). At 3dpf, in *Tg(2P-EIand1*:*eGFP)* line as in *Tg(2Pand1*:*eGFP)*, reporter expression occurs within the MFF ectoderm and migrating mesenchymal cells (Fig 1C). Note that in both *Tg(2Pand1*:*eGFP)* and *Tg(2P-EIand1*:*eGFP)*, expression in the mesenchymal cells is masked by that of the overlying ectoderm, however we have previously shown eGFP expression in both cell layers of the *Tg(2Pand1*:*eGFP)* transgenic line by confocal microscopy [14]. Mesenchymal cells expressing *and1* can be morphologically distinguished from *and1*-expressing ectodermal cells in that the former are elongated and branched [14] (Fig 1B’). Ectodermal cells are more hexagonal in shape [14] (Fig 1B’). We previously showed that in *Tg(epi*.*and1-βG*:*eGFP)*, the *epi* region (located at positions -1117 to -967) and a minimal human beta-globin (βG) promoter (Fig 1A) drive reporter expression specifically within the MFF ectoderm in 3dpf larvae (Fig 1D) [14]. Lastly, in *Tg(2PΔepi*.*and1*:*eGFP)*, the *2P* region excluding the *epi* region (Fig 1A) drives reporter expression specifically within the mesenchymal tissue located in the fin fold of 3dpf larvae (Fig 1E).

As zebrafish reach 30dpf, the lepidotrichia emerge, allowing the MFF at the posterior part of the larva to transition from a rounded, blunt shape to a bi-lobed structure that will become the caudal fin [25]. In *Tg(2P-EIand1*:*eGFP)*, reporter expression is brightly observed in the ectoderm that lines the edges of the developing caudal fin, and in the fin rays (Fig 1G). In the *Tg(2PΔepi*.*and1*:*eGFP)* transgenic line, reporter expression is solely observed in the fin rays (Fig 1I). In *Tg(2Pand1*:*eGFP)*, reporter expression is present within the fin rays and ectodermal tissue, but is comparably fainter than that of *Tg(2P-EIand1*:*eGFP)* and *Tg(2PΔepi*.*and1*:*eGFP)* (Fig 1F, 1G and 1I). The *Tg(epi*.*and1-βG*:*eGFP)* transgenic line no longer drives detectable reporter expression at 30dpf and throughout the remainder of development to adulthood (Fig 1H, 1L and 1P).

**Fig 2:**
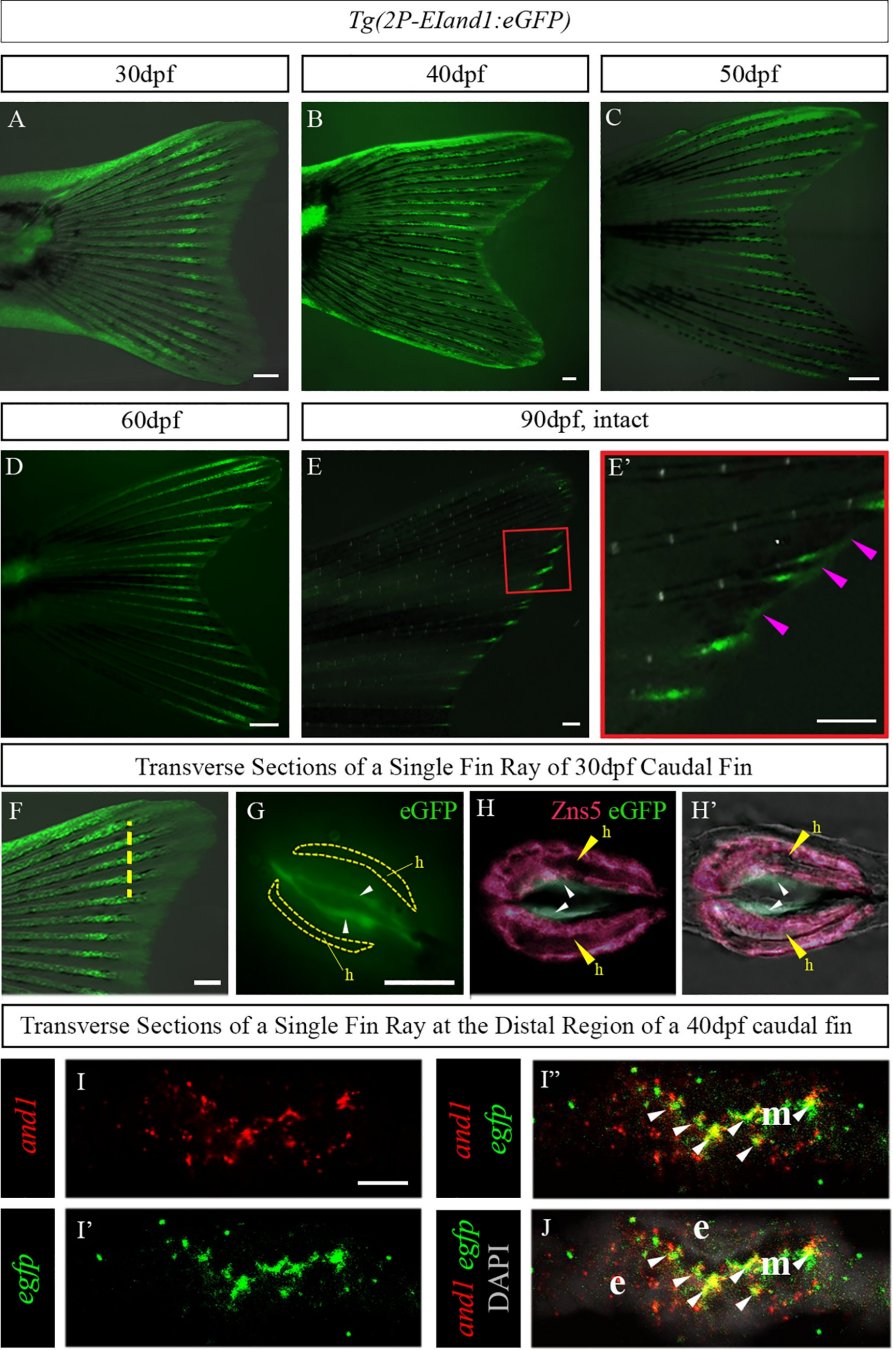
Reporter expression in the developing caudal fin of *Tg(2P-EIand1*:*eGFP)*. (A) Reporter expression of *Tg(2P-EIand1*:*eGFP)* (25 fish were analysed) occurs within the ectodermal tissue and fin rays along the proximal-distal axis of the developing caudal fin. It is notably brighter as it approaches the distal region at 30dpf. (B) Reporter expression occurs along the proximal-distal axis in the fin rays and is beginning to approach the distal edge of the fin where ectodermal-specific expression is present at 40dpf. (C, D) At 50dpf and 60dpf, eGFP expression in the fin rays distalizes and is present in the epithelial tissue located at the very distal regions of the fin. (E, E’) Reporter expression only occurs at the distal tips of the fin rays and at the distal edge of the interrays (pink arrowheads). (E’) close-up on panel E. (F) Transverse sections from a 30dpf caudal fin were obtained in the region indicated by the yellow dotted line. (G) Immunostaining showing eGFP expression (white arrowheads) localized deep to the hemirays (h; delineated by yellow line) (n = 6). Immunostaining for Zns5, which is a pan-osteoblast marker, labelling osteoblasts surrounding the hemirays (h; yellow arrowheads) and eGFP (small white arrowheads) in *Tg(2P-EIand1*:*eGFP)* on transverse cryosections of 30dpf larvae (n = 6). (H) eGFP is localized in layers of cells deep and adjacent to osteoblast layers lining the hemirays (h). (H’) Merge on bright field. Double fluorescence *in situ* hybridization (n = 12) for *and1* (I) and *eGFP* of *Tg(2P-EIand1*:*eGFP)* (I’). (I”) Merge between red and green showing colocalization between *and1* and *egfp* (white arrowheads). (J) Merge with DAPI showing colocalization occurring within the fin ray mesenchymal tissues. Note: Any expression observed outside of the fin ray mesenchymal compartment is background staining. n = # of fish from which sections were obtained. Scale bars: A-E, E’ = 200μm, F = 100μm, G, H-H’ = 50μm and I-I”, J = 10μm.

As zebrafish reach 60dpf, they are considered to have transitioned from the larval to juvenile stage; the MFF has fully undergone resorption and the dorsal, ventral and caudal fins are present [25]. At 60dpf, reporter expression is absent in *Tg(2Pand1*:*eGFP)* (Fig 1J), while *Tg(2P-EIand1*:*eGFP)* and *Tg(2PΔepi*.*and1*:*eGFP)* fish continue to have reporter expression at the distal region of the caudal fin (Fig 1K and 1M). In *Tg(2P-EIand1*:*eGFP)*, reporter expression is strong in the interray tissue and in the rays (Fig 1K; see also Fig 2D–2D’). In *Tg(2PΔepi*.*and1*:*eGFP)*, expression still occurs only in the fin rays to levels that are detectable by epifluorescence microscopy; however visually, levels of fluorescence are lower than *Tg(2P-EIand1*:*eGFP)* (Fig 1K and 1M). These expression patterns of all *and1* reporter lines observed at 60dpf are similar to those at >90dpf (Fig 1N–1Q).

The absence of reporter expression in *Tg(2Pand1*:*eGFP)* and *Tg(epi*.*and1-βG*:*eGFP)* reporter lines in contrast to the presence of reporter expression observed in *Tg(2PΔepi*.*and1*:*eGFP)* lines throughout development to adulthood suggests that the *epi* region may contain a repressor that is active during adulthood. Furthermore, the presence of strong reporter expression in *Tg(2P-EIand1*:*eGFP)* lines, despite the inclusion of this potential *epi* repressor, suggests that the *EI* region may contain enhancers required for the maintenance of reporter expression throughout development. Overall, the variation in reporter expression seen throughout development in the aforementioned transgenic lines suggests that the *cis*-acting regulation of *and1* differs between embryonic development and later development towards adulthood. The details regarding these changes in *and1* regulation will be discussed in upcoming sections.

### Reporter expression of *Tg(2P-EIand1*:*eGFP)* recapitulates endogenous *and1* expression

#### Reporter expression in *Tg(2P-EIand1*:*eGFP)* throughout development

During the transition from the larval to juvenile stage, reporter expression in *Tg(2P-EIand1*:*eGFP)* occurs in the interray tissue and along the proximal-distal axis of the fin rays (Fig 2A–2C). As development progresses towards adulthood, reporter expression gradually becomes restricted to the more distal regions of the growing caudal fin, where actinotrichia fibres are located (Fig 2B–2D) [12]. At adulthood, reporter expression is fully maintained at the distal tips of the fin rays as well as along the distal epithelial tissue that lines the entire fin (Fig 2E–2E'). In order to determine the specific location of eGFP(+) cells, double immunostaining on cryosections was performed for eGFP and Zns5, a pan-osteoblast marker [26]. Reporter eGFP expression, in transverse cryosections through a single fin ray, is localized within the cells internal to the osteoblast layers that surround the hemirays (Fig 2F, 2G and 2H–2H’).

In order to determine if the distal restriction of reporter expression in *Tg(2P-EIand1*:*eGFP)* corresponds to a distal restriction in endogenous *and1* expression and actinotrichia formation during the early juvenile stage, we performed an *in situ* hybridization (ISH) for *eGFP* and immunostaining for And1/2 [13]. Transverse cryosections were obtained from the distal and proximal (midway down the fin) regions of the developing caudal fin at 40dpf. Both *eGFP* and *and1* mRNA are absent in the sections obtained from the proximal region of the caudal fin (S1A–S1A' and S1B–S1B' Fig). Conversely, *eGFP* and *and1* are present in the fin ray mesenchymal tissue in sections obtained from the distal region of the caudal fin (S1C–S1C' and S1D–S1D' Fig). Similarly, immunostaining for And1/2, which allows to visualize the actinotrichia, shows that the actinotrichia are absent in the more proximal regions of the caudal fin and present in the distal regions of the caudal fin (S1E–S1E' and S1F–S1F' Fig). In order to confirm that *eGFP* and *and1* colocalize, double fluorescence *in situ* hybridization (FISH) experiments were performed on proximally and distally located transverse sections with *eGFP* and *and1* probes. Indeed, the expression patterns of *eGFP* and *and1* co-localize within the fin ray mesenchymal tissue of the developing caudal fin of fish reaching the juvenile stage (Fig 2I–2I’ and 2J).

#### Reporter expression in *Tg(2P-EIand1*:*eGFP)* throughout adult fin regeneration

**Fig 3:**
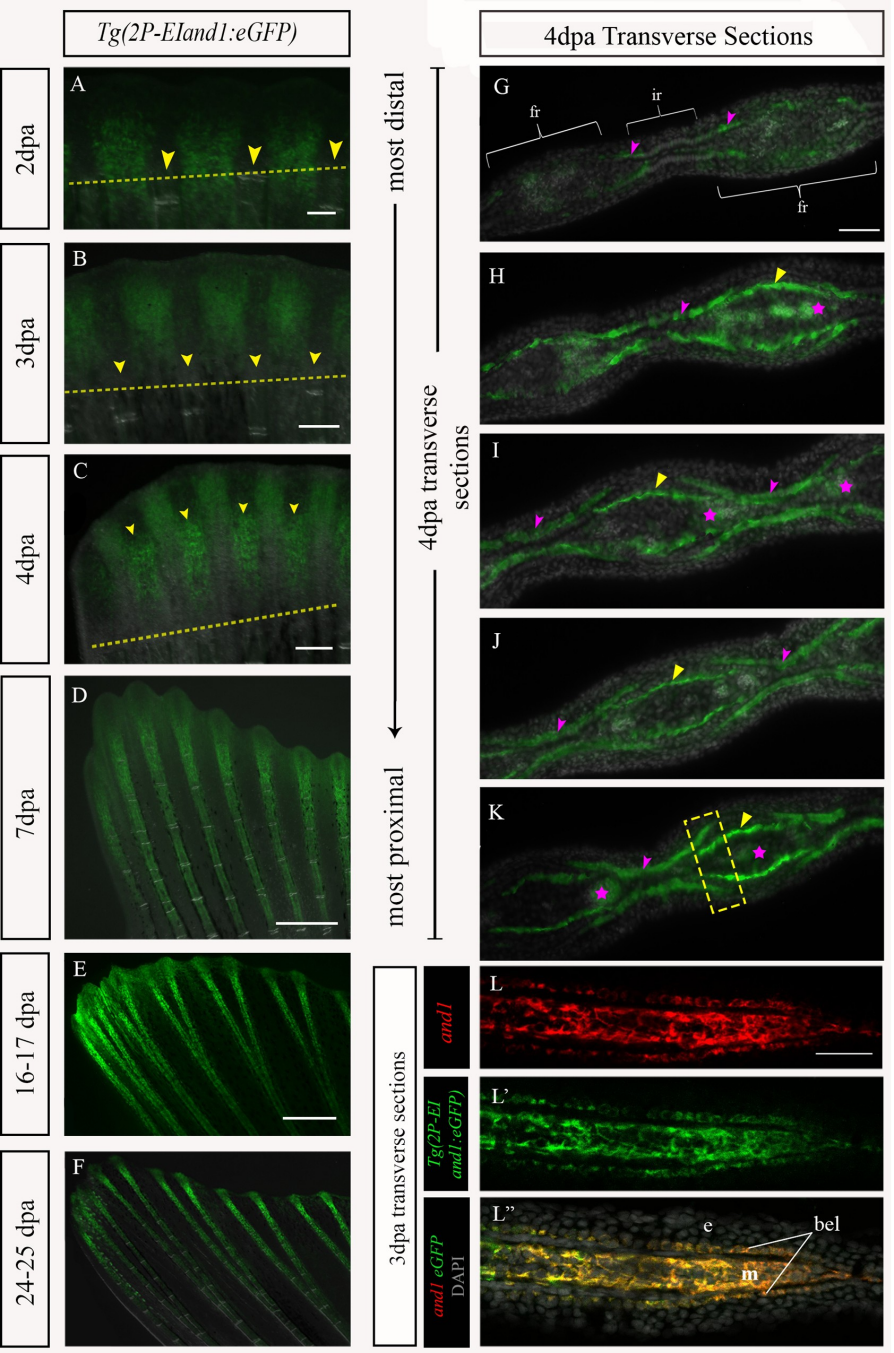
*Tg(2P-EIand1*:*eGFP)* recapitulates *and1* expression throughout regeneration. (A-F) *In vivo* time course analysis of eGFP reporter expression during fin regeneration in *Tg(2P-EIand1*:*eGFP)* (n = 12). (A-B) At 2dpa and 3dpa, reporter expression is brightly observed only in the interray tissue of *Tg(2P-EIand1*:*eGFP)*. Yellow arrowheads indicate location of fin ray tissue. Yellow dotted line delineates amputation plane. (C-D) At 4dpa and 7dpa, fin ray-specific reporter expression occurs along the proximal-distal axis and is as equally bright as that of interray expression in *Tg(2P-EIand1*:*eGFP)*. (E-F) Fin ray-specific reporter expression becomes distally restricted in *Tg(2P-EIand1*:*eGFP)*. (G-K) Immunostaining for eGFP in *Tg(2P-EIand1*:*eGFP)* on consecutive transverse cryosections of 4dpa fin regenerate. (G) In the most distal region of the regenerate, reporter expression faintly occurs in the basal epithelial layer (pink arrowheads) surrounding the hemirays. The fin ray and interray are indicated by white brackets. (H-K) As sections progress proximally, reporter expression within the basal epithelial layer (pink arrowheads) is restricted towards the interray region. Reporter expression within the mesenchymal tissue is indicated by yellow arrowheads. (I-K) Reporter expression within basal epithelial layer is absent in middle regions of hemirays and overlaps with mesenchymal-specific expression in regions of hemirays that are closer to interrays (indicated by dotted yellow box in Panel K). Pink stars indicate autofluorescence from blood vessels. Double fluorescence *in situ* hybridization (n = 8) on longitudinal sections (proximal to the left and distal to the right hand sides of the panels) of 3dpa fin regenerate for (L) *and1* and (L’) *eGFP* of *Tg(2P-EIand1*:*eGFP)*. (L”) Merge with DAPI staining of cell nuclei. fr: fin ray, ir: interray, m: mesenchyme; bel: basal epithelial layer; e: epithelium. n = # of fish of which fins were sectioned and on which given experiment was performed. Scale bars: A = 100μm, B-F = 200μm, G-K = 50μm, L-L” = 50μm.

During the early steps of adult fin regeneration, reporter expression is first observed in the interray tissue of the regenerate at 2dpa (Fig 3A). At 3dpa, reporter expression is predominantly in the interray tissue (Fig 3B). At 4dpa to 7dpa, reporter expression is brightly observed along the proximal-distal axis in the fin rays, while interray-specific reporter expression remains within the distal regions of the regenerate (Fig 3C and 3D). After ~7-9dpa, reporter expression within the fin rays and interray tissue gradually distalizes (Fig 3E and 3F). To determine the specific cell layers in which eGFP(+) cells occur, immunostaining for eGFP was performed on consecutive transverse cryosections of 4dpa regenerates (Fig 3G–3K). In the distal-most region of the regenerate, eGFP expression only occurs in the basal epithelial layers of the interrays (Fig 3G). As the sections progress more proximally, eGFP expression is present in distinct layers of cells of the interrays and fin rays (Fig 3H). In the interrays, eGFP is only found in the basal epithelial layer (Fig 3H–3K). In the middle region of the fin rays (Fig 3H–3K), eGFP is only present in the mesenchymal tissue layers. However, in the lateral parts of the hemiray that are closer to the interrays, eGFP is present in both the basal epithelial layer and mesenchymal tissue (Fig 3H–3K).

In order to compare endogenous *and1* expression with *Tg(2P-EIand1*:*eGFP)* during adult fin regeneration, double FISH experiments for *and1* and *eGFP* were performed on consecutive longitudinal cryosections of 3dpa fin regenerates. *And1* and *eGFP* expression co-localize in the fin ray mesenchyme and in the basal epithelial layer along the proximal-distal axis of the regenerate (Fig 3L–3L”). Overall, the observed co-localization of *eGFP* and endogenous *and1* expression throughout development and in adult fin regeneration confirms that the *Tg(2P-EIand1*:*eGFP)* line recapitulates endogenous *and1* expression.

### The *epi3* site within the *epi* region contains an interray basal epithelial-specific enhancer and early mesenchymal-specific repressor for adult fin regeneration

The absence of reporter expression in the intact fins of *Tg(2Pand1*:*eGFP)* in contrast to the presence of fin ray-specific expression in *Tg(2PΔepi*.*and1*:*eGFP)* during adulthood suggests that the *epi* region may contain a repressor sequence (Fig 1N and 1Q). To further analyze the activity of this potential repressor sequence, we performed a comparative time course analysis of reporter expression in *Tg(2P-EIand1*:*eGFP)*, *Tg(2Pand1*:*eGFP)*, and *Tg(2PΔepi*.*and1*:*eGFP)* during fin regeneration. The *Tg(epi*.*and1-βG*:*eGFP)* line lacks reporter expression throughout regeneration (S2 Fig) and was, therefore, excluded from comparative analyses.

**Fig 4:**
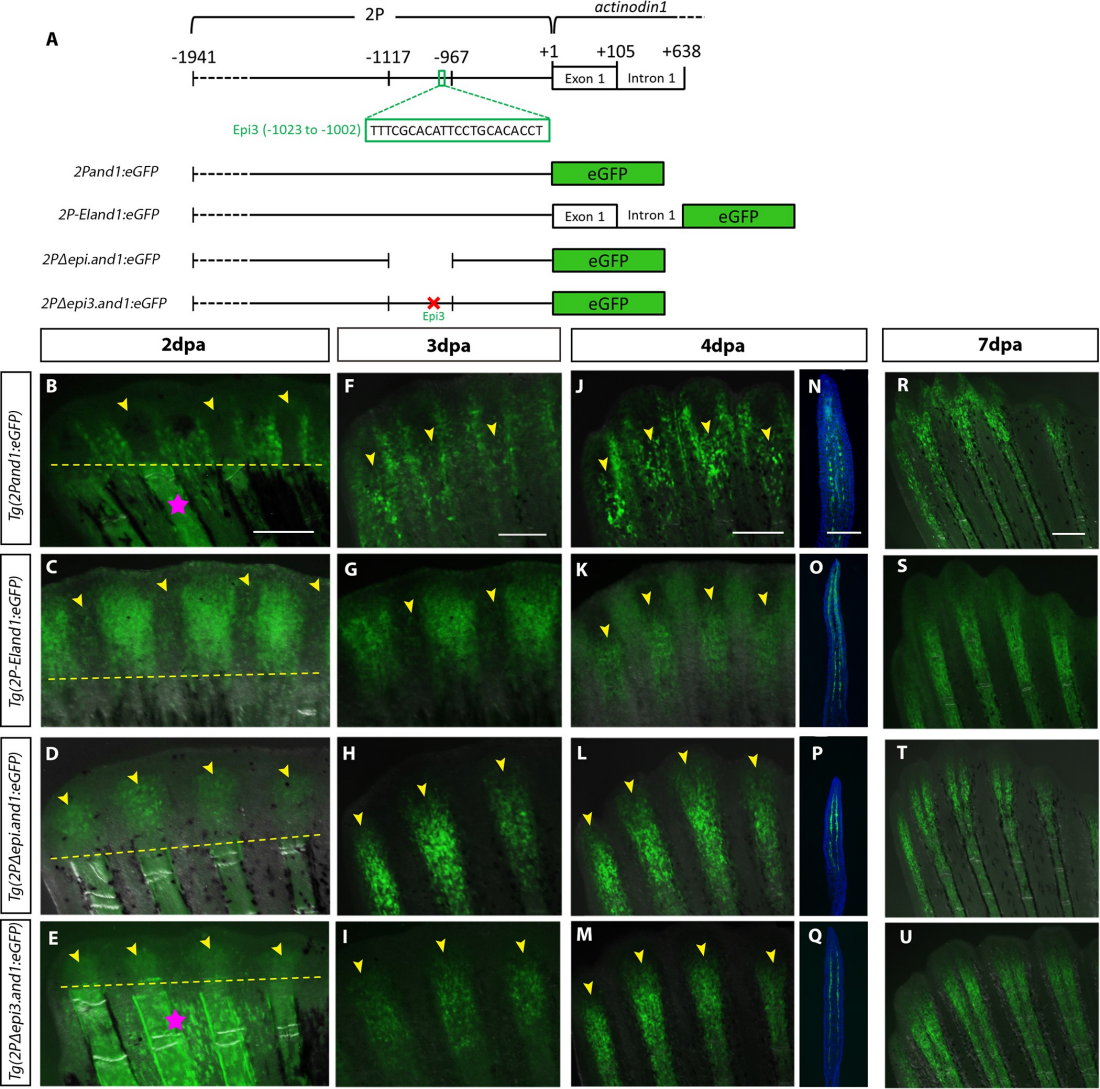
The *epi3* site within the *epi* region contains a mesenchymal-specific repressor that is active early in regeneration. (A) Schematic representation of constructs. *In vivo* time course analysis of reporter expression in *Tg(2Pand1*:*eGFP)* (n = 7) (B, F, J, R), *Tg(2P-EIand1*:*eGFP)* (n = 12) (C, G, K, S), *Tg(2PΔepi*.*and1*:*eGFP)* (n = 11) (D, H, L, T), and *Tg(2PΔepi3*.*and1*:*eGFP)* (n = 13) (E, I, M, U) during fin regeneration. Fluorescent images are merged with bright field images. (N-Q) Immunostaining for eGFP on longitudinal cryosections of 4dpa fin regenerates. (B-E) At 2dpa, *Tg(2Pand1*:*eGFP)* and *Tg(2P-EIand1*:*eGFP)* only have interray epithelial-specific expression while *Tg(2PΔepi*.*and1*:*eGFP)*, and *Tg(2PΔepi3*.*and1*:*eGFP)* have fin ray mesenchymal-specific expression. (F, G) At 3dpa, *Tg(2Pand1*:*eGFP)* and *Tg(2P-EIand1*:*eGFP)* have bright interray epithelial-specific expression and faint fin ray mesenchymal-specific expression. (H, I, L, M) In *Tg(2PΔepi*.*and1*:*eGFP)* and *Tg(2PΔepi3*.*and1*:*eGFP)*, strong fin ray mesenchymal-specific expression is observed. (J, N, R) At 4dpa and 7dpa, fin ray mesenchymal- and basal epithelial-specific expression in *Tg(2Pand1*:*eGFP)* is faint and patchy. (K-M, O-Q, S-U) Reporter expression in *Tg(2P-EIand1*:*eGFP)* is bright in the interray, while fin ray mesenchymal-specific expression is just as bright as that of *Tg(2PΔepi*.*and1*:*eGFP)* and *Tg(2PΔepi3*.*and1*:*eGFP*). Time course analysis was done on one line for each construct; n = # of fish/ line. Pink stars in B and E indicate autofluorescence from white pigment cells and blood vessels. Yellow arrowheads indicate fin ray regenerative tissue. Amputation plane is delineated by yellow dotted line. Scale bars = 200μm.

As previously shown in the proximal regions of fin regenerates, reporter expression occurs in the basal epithelial layer of the interrays and in the mesenchymal cells of the fin rays in *Tg(2P-EIand1*:*eGFP)* (Fig 3I–3K). At 2dpa, *Tg(2Pand1*:*eGFP)* and *(Tg(2P-EIand1*:*eGFP)* reporter expression is first seen in the regenerative interray tissue (Fig 4A–4C). In contrast, *Tg(2PΔepi*.*and1*:*eGFP)* is the only transgenic line among the three to exhibit reporter expression within the fin ray regenerative tissue (Fig 4A and 4D). At 3dpa, fin ray mesenchymal-specific reporter expression is detectable in *Tg(2Pand1*:*eGFP)* and *Tg(2P-EIand1*:*eGFP)*, but is fainter than that of *Tg(2PΔepi*.*and1*:*eGFP)* (Fig 4F–4H). It is only at 4dpa when fin ray mesenchymal-specific expression in *Tg(2Pand1*:*eGFP)* and *Tg(2P-EIand1*:*eGFP)* is comparable to that of *Tg(2PΔepi*.*and1*:*eGFP)* (Fig 4J–4M). Immunostaining for eGFP on longitudinal sections of these transgenic lines confirm GFP expression in ray mesenchyme at 4 dpa (Fig 4N–4Q). At 7dpa, all four transgenic reporter lines have fin ray mesenchymal-specific reporter expression, although that of *Tg(2Pand1*:*eGFP)* is consistently fainter or patchier than that of *Tg(2P-EIand1*:*eGFP)* and *Tg(2PΔepi*.*and1*:*eGFP)* (Fig 4R–4U). In summary, reporter expression is faintly observed in *Tg(2P-EIand1*:*eGFP)* and *Tg(2Pand1*:*eGFP)* within the fin ray mesenchymal tissue up to 4dpa, while *Tg(2PΔepi*.*and1*:*eGFP)* displays strong reporter expression in the same region at the same time points. Thus, the removal of *epi* in *Tg(2PΔepi*.*and1*:*eGFP)* results in notable fin ray mesenchymal-specific reporter expression during the early stages of regeneration, suggesting that *epi* contains an early fin ray mesenchymal-specific repressor. It was also noted that, in adulthood and fin regeneration, the *Tg(2PΔepi*.*and1*:*eGFP)* line also completely lacks interray basal epithelial-specific reporter expression (Figs 1Q, 4D, 4H, 4L and 4T), suggesting that the previously identified embryonic/early larval ectodermal enhancer within the *epi* region [14] also functions to enhance *and1* expression within the basal epithelial layer of the adult fin regenerate.

Previous TRANSFAC analysis of the *epi* region allowed identification of four clusters of putative binding sites. These sites, termed *epi1-4*, were originally identified and their potential activity was analyzed during embryogenesis [14]. Four transgenic reporter lines, in which each of the four *epi* sites of the constructs was deleted or substituted via site-directed mutagenesis, were generated; three mutations consisted of deletions (*epi1-3)* from the *2P* region and one consisted of a 2bp substitution (*epi4*) [14]. These same transgenic lines were also analysed in order to characterize their potential activity in adult fin regeneration. Of these four transgenic lines, *Tg(2PΔepi3*.*and1*:*eGFP)* has reporter expression that is identical to that of *Tg(2PΔepi*.*and1*:*eGFP)* throughout regeneration in that there is only fin ray mesenchymal-specific expression and a complete absence of interray basal epithelial-specific expression (Fig 4D, 4E, 4H–4I, 4L, 4M, 4P, 4Q, 4T and 4U). Therefore, evidence suggests that the *epi3* site contains an early mesenchymal-specific repressor. In addition, the complete absence of interray-specific expression in *Tg(2PΔepi3*.*and1*:*eGFP)* throughout regeneration suggests that the previously identified larval fin fold ectodermal enhancer is required for interray basal epithelial-specific reporter expression.

### The *EI* region contains an alternative promoter for *and1* expression during adulthood

**Fig 5:**
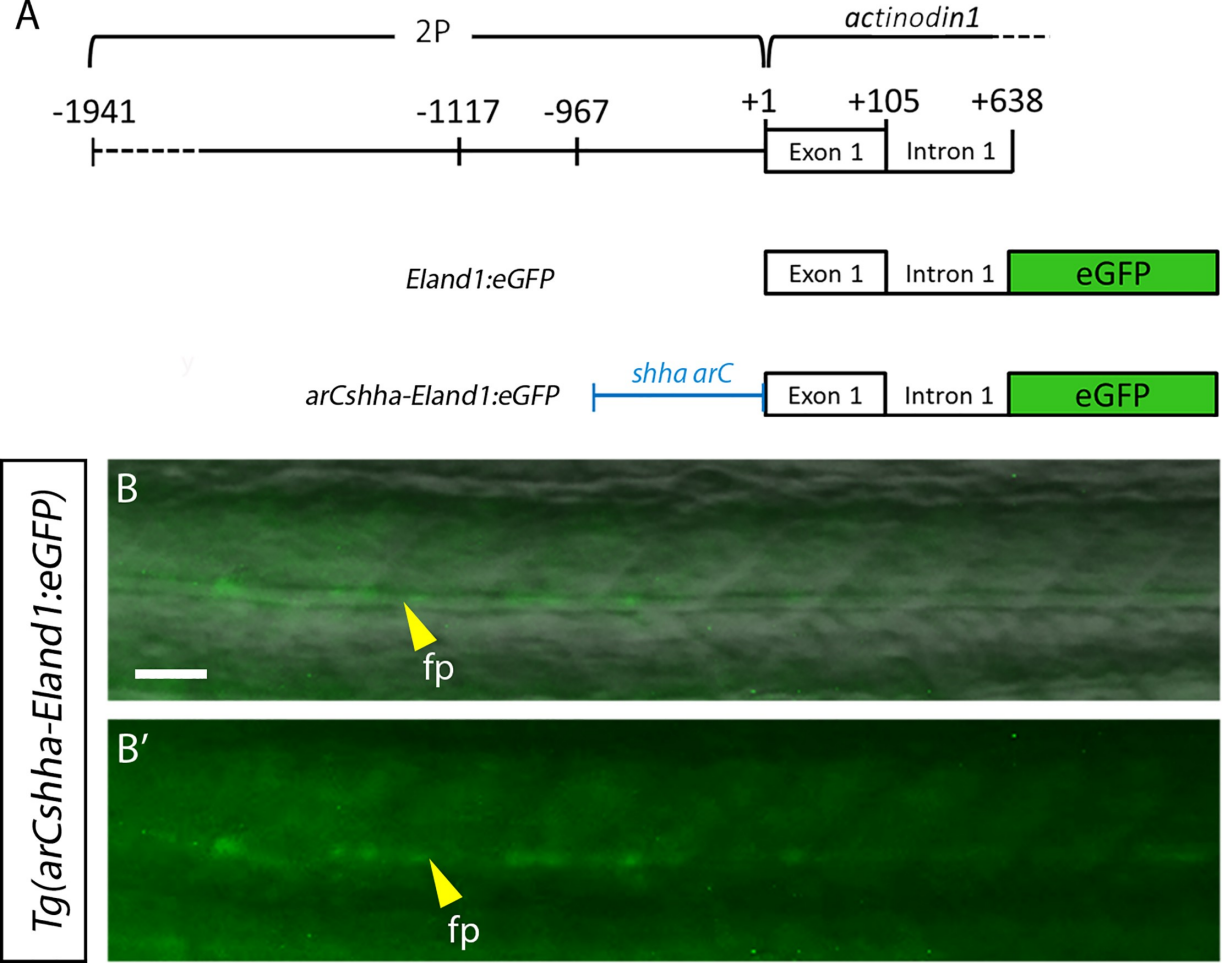
*EI* contains an alternative promoter. (A) Schematic representation of construct. (B-B’) 1dpf *Tg(arCshha-EIand1*:*eGFP)* F1 generation embryos showing *eGFP* expression in the floor plate cells (n = 15 fish, 1 line). (B) Merge with bright field image. (B’) Fluorescent image only. fp: floor plate. Scale bar: B-B’ = 25μm.

Compared with *Tg(2Pand1*:*eGFP)*, the *Tg(2P-EIand1*:*eGFP)* line has very strong reporter expression in adult intact fins and during adult fin regeneration; this suggests that the *EI* region contains enhancers and an additional promoter required for adequate transgene expression during adulthood. To test for the presence of a promoter in *EI*, the *EI* region was cloned into an *eGFP* reporter construct containing a *sonic hedgehog a* (*shha) arC* enhancer element (Fig 5A) and zebrafish transgenic lines were generated with this construct. In the presence of a promoter, the *arC* enhancer can drive weak reporter expression in the floor plate and notochord regions of zebrafish larvae [27]. The *Tg(arCshha-EIand1*:*eGFP)* embryos consistently exhibit faint, but detectable reporter expression within the floor plate and notochord at 1dpf (Fig 5B–5B’), which suggests the presence of a functional alternative promoter within the *EI* region. Taking into account the disappearance of both fin interray epithelial- and mesenchymal-specific reporter expression in *Tg(2Pand1*:*eGFP)* compared with *Tg(2P-EIand1*:*eGFP)*, it is possible that this alternative promoter may be required for both epithelial- and mesenchymal-specific *and1* expression during adulthood.

### The *EI* region may contain an adult tissue transcriptional enhancer

When comparing the expression patterns of the *Tg(2Pand1*:*eGFP)* and *Tg(2P-EIand1*:*eGFP)* lines during the embryonic stage, strong reporter expression in the mesenchymal and ectodermal cells of the MFF was observed (Fig 1B and 1C). However, in adulthood, this reporter expression only strongly persists in *Tg(2P-EIand1*:*eGFP)* (Fig 1K and 1O) and nearly disappears in *Tg(2Pand1*:*eGFP)* (Fig 1J and 1N); this suggests that the inclusion of *EI* significantly increases reporter expression. During regeneration, reporter expression in *Tg(2Pand1*:*eGFP)* occurs in both the interray basal epithelial layer and fin ray mesenchymal tissue (Fig 4B, 4F, 4J, 4N and 4R); however, its expression is fainter and patchier than the reporter expression observed in *Tg(2P-EIand1*:*eGFP)* (Fig 4C, 4G, 4K, 4O and 4S). This difference in strength of reporter expression between *Tg(2Pand1*:*eGFP)* and *Tg(2P-EIand1*:*eGFP)* suggests that the enhancer functions in the maintenance of *and1* expression throughout development and adulthood, and in boosting *and1* expression throughout regeneration.

**Fig 6:**
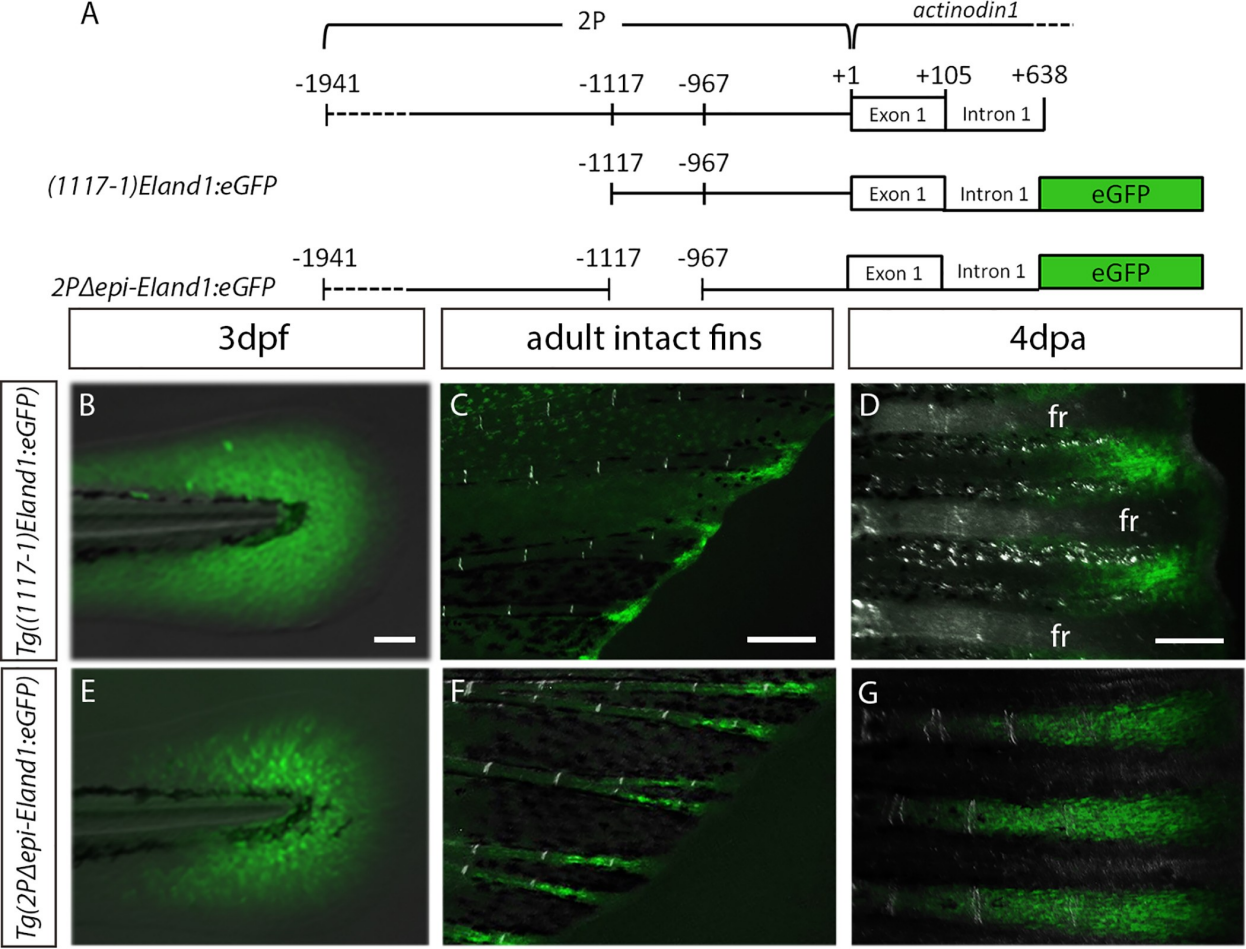
*EI* contains an adult tissuetranscriptional booster. *In vivo* time course analysis of reporter expression of *Tg((1117–1)EIand1*:*eGFP)* (N = 3; n = 35) (B-D) and *Tg(2PΔepi-EIand1*:*eGFP)* (N = 3; n = 31) (E-G), at 3dpf (B, E), in intact fins at 90dpf (C, F), and at 4dpa (D, G) during fin regeneration. Note: adult fins are oriented in the proximal to distal direction (left to right). (B-D) *Tg((1117–1)EIand1*:*eGFP*) expression only occurs in the ectodermal/basal epithelial tissue; (E-G) *Tg(2PΔepi-EIand1*:*eGFP)*, only mesenchymal tissue. fr: fin ray. N = number of lines found; n = total number of fish analysed. Scale bars: B, E = 25μm, C, F = 200μm and D, G = 200μm.

In order to test whether or not the inclusion of *EI* could enhance interray basal epithelial-specific reporter expression driven by the *epi* region, the region continuous from the beginning of *epi* to the end of the first intron (positions -1117 to +638) was cloned into an *eGFP* construct, yielding a construct named *(1117–1)EIand1*:*eGFP* (Fig 6A). It is important to note that the region spanning from positions -967, (3’ end of *epi*), to +1 was found to drive no significant transgene expression on its own during embryonic development [14]. At 3dpf, ectodermal-specific reporter expression is present in the MFF (Fig 6B). In adulthood, two out of three *Tg((1117–1)EIand1*:*eGFP)* lines (n = 5–6 fish per line) show strong reporter expression within the interray tissue at the distal regions of the intact fin (Fig 6C). During regeneration, strong reporter expression within the interray tissue is also observed (Fig 6D and S3 Fig). This interray-specific reporter expression mimics that of the *Tg(2P-EIand1*:*eGFP)* line, where it also remains confined within the distal regions of the regenerate (S3G–S3L Fig). Immunostaining for eGFP on transverse cryosections of 4dpa regenerates of *Tg((1117–1)EIand1*:*eGFP)* show that transgene expression is specifically located in the basal epithelial layer of the interray tissue and absent in the middle regions of the hemirays (S4 Fig). Taken together, we were able to successfully create a transgenic line in which reporter expression is specific to the basal epithelial layer and is maintained throughout adulthood, while showing that the *EI* region of *and1* contains a strong transcriptional enhancer that is required for adequate reporter expression to be observed in adulthood and fin regeneration.

Finally, three transgenic lines were generated for the construct *2PΔepi-EIand1*:*eGFP* in which the *2P-EI* region excluding the *epi* region drive reporter expression (Fig 6A). As expected, at 3dpf, reporter expression is observed specifically in the elongated- and branched-shaped mesenchymal cells of the MFF (Fig 6E). However, in adulthood, reporter expression within the fin ray mesenchymal tissue was noticeably and consistently bright within the intact fins and within 4dpa regenerates in all three *Tg(2PΔepi-EIand1*:*eGFP)* lines (n = 10–15 fish per line) that we generated (Fig 6F and 6G). Indeed, although the *Tg(2PΔepi*.*and1*:*eGFP)* line still has reporter expression at the distal tips of the fin rays, its expression tends to be weaker and patchier than that of *Tg(2P-EIand1*:*eGFP)* and is not consistently present in all of the fin rays of all fish that have surpassed the 90dpf time point (S5 Fig). It appears that the inclusion of *EI* is still required in order to fully recapitulate *and1* expression in the fin ray mesenchymal tissue of adult intact fins.

## Discussion

**Fig 7:**
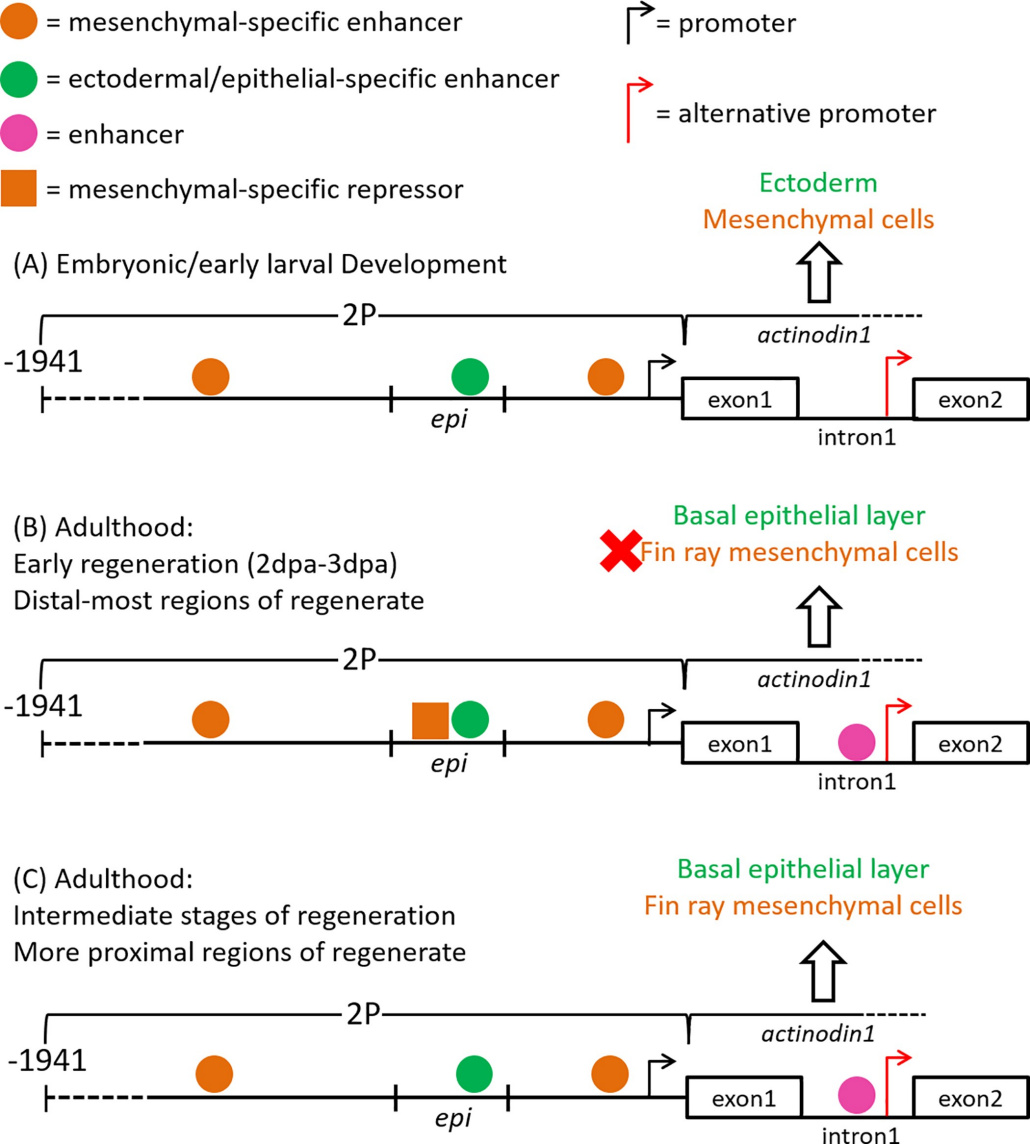
Regulation of *actinodin1* during embryonic and early larval development, and during adult fin regeneration. (A) During early larval development, the ectodermal-specific enhancer binds to the *epi* region, while mesenchymal-specific enhancers bind to the *2P* regions outside of *epi*. These enhancers, together, promote *and1* expression in the ectoderm and migrating mesenchymal cells of the fin fold. (B) During the early stages of regeneration and in the distal-most regions of the regenerating fin, fin ray mesenchymal-specific *actinodin1* expression is inhibited, perhaps through the binding of a strong repressor within the *epi* region. In parallel, strong *actinodin1* expression occurs within the basal epithelial layer due to the binding of an epithelial-specific enhancer at the *epi* region and perhaps at various enhancer sequences within the *EI* region. The repressor effects at the level of *epi* appear to override intron-mediated enhancement. (C) During the intermediate stages of regeneration, within the more proximal regions of the regenerate, the absence of the *epi*-specific repressor and the persistence of intron-mediated enhancement allows for strong *actinodin1* expression in the basal epithelial layer and in the fin ray mesenchyme.

Time course analysis of several *and1* reporter lines throughout development revealed that there is a change in *and1* regulation as the zebrafish reaches adulthood. During larval development, the *Tg(2Pand1*:*eGFP)* and the *Tg(2P-EIand1*:*eGFP)* lines exhibit strong reporter expression in the migrating mesenchymal cells of the MFF and in the overlying ectoderm. As for the *Tg(epi*.*and1-βG*:*eGFP)* and *Tg(2PΔepi*.*and1*:*eGFP)* lines, reporter expression is observed in only the ectoderm or fin fold mesenchymal tissue, respectively. As the zebrafish transition towards the juvenile stage, reporter expression in *Tg(2Pand1*:*eGFP)* and in *Tg(epi*.*and1-βG*:*eGFP)* disappears, while that of *Tg(2P-EIand1*:*eGFP)* and *Tg(2PΔepi*.*and1*:*eGFP)* remains at the distal region of the growing caudal fin. This stage-dependent disappearance of reporter expression in *Tg(2Pand1*:*eGFP)* and *Tg(epi*.*and1-βG*:*eGFP)* in contrast to the persistence of that of *Tg(2PΔepi*.*and1*:*eGFP)* lines suggests the existence of a repressor that is located within the *epi* region and that may have regulatory functions during adulthood. As we further investigated the role of this potential repressor, we found that a site, termed *epi3*, functions to suppress mesenchymal-specific expression during the early stages of fin regeneration and to enhance basal epithelial-specific expression. Furthermore, we remarked that reporter expression in the *Tg(2P-EIand1*:*eGFP)* line is still strongly maintained in adulthood. This observation suggests that (1) the *Tg(2P-EIand1*:*eGFP)* line can fully report *and1* expression throughout development and adulthood, and (2) the *EI* region may contain enhancers and an alternative promoter that are essential to the maintenance of *and1* expression throughout development (Fig 7).

### The *Tg(2P-EIand1*:*eGFP)* line reports *and1* expression in adulthood and regeneration

During the intermediate stages of larval development (30dpf), reporter expression in the *Tg(2P-EIand1*:*eGFP)* line is strong in the ectoderm and along the proximal-distal axis in the mesenchymal tissue enclosed within the fin rays. This observation may reflect the presence of actinotrichia along the developing caudal fin in order to provide mechanical support and to serve as a scaffold for mesenchymal cell migration during morphogenesis [4, 9, 13]. As development progresses towards adulthood, reporter expression gradually distalizes until it remains restricted at the distal tips of the fin rays and distal epithelial tissue lining the adult fin. In adults, actinotrichia are located at the distal tip of each lepidotrichia in the intact fin [1]. Furthermore, they have also been observed to remain distally restricted at the tip of each fin ray throughout fin development and regeneration [4, 12]. Overall, the spatial and temporal dynamics of *Tg(2P-EIand1*:*eGFP)* reporter expression throughout development matches that of actinotrichia formation. Moreover, endogenous *and1* expression spatially and temporally correlates with actinotrichia formation [13], which further suggests that the *Tg(2P-EIand1*:*eGFP)* line recapitulates endogenous *and1* expression throughout development.

In the early to intermediate stages of regeneration (3dpa-7dpa), *Tg(2P-EIand1*:*eGFP)* reporter expression colocalizes with endogenous *and1* expression within the basal epithelial layer of the interray tissue and the mesenchymal tissue located deep to the osteoblasts layers. The expression pattern of *Tg(2P-EIand1*:*eGFP)* correlates with the presence of actinotrichia, which are found to be displaced by the osteoblasts into the mesenchymal compartment [11]. Interestingly, *Tg(2P-EIand1*:*eGFP)* expression occurs along the proximal-distal axis within the fin rays, similarly to the actinotrichia fibers. The presence of actinotrichia in these regions supports a role in cell migration as suggested by recent findings that show the cytoplasm of blastemal cells and differentiating cells engulfing nearby actinotrichia fibers [11]. The similarity between the expression patterns of *Tg(2P-EIand1*:*eGFP)* and endogenous *and1* expression suggests that the *Tg(2P-EIand1*:*eGFP)* line can recapitulate endogenous *and1* expression throughout regeneration.

### The *EI* region can be used to drive tissue-specific expression in adulthood

During larval development, the omission or addition of the first intron and exon of *and1*, (collectively known as *EI*), in the transgenic constructs yields no difference in MFF reporter expression. In contrast, the addition of *EI* shows a notable difference in levels of fluorescence during adulthood. As these differences in strength of reporter expression are visible only as the zebrafish reaches adulthood, it is possible that the regulatory elements within *EI* are mainly activated during adulthood. A summary of activity of all reporter constructs during larval development and adulthood is contained in Table 1.

**Table 1:** Change in expression of several *and1* reporter lines towards adulthood.

	Expression during Embryonic Development	Adult Intact Fins
***Tg(2Pand1*:*EGFP)***	ectoderm + mesenchyme	None
***Tg(2P-EIand1*:*eGFP)***	ectoderm + mesenchyme	Basal epithelial layer in interray + fin ray mesenchyme
***Tg(Epi*.*and1-*** *βG*:*EGFP****)***	ectoderm	None
***Tg((1117–1)EIand1*:*eGFP)***	ectoderm	Basal epithelial layer of interray
***Tg(2P****Δ****epi*.*and1*:*EGFP)***	mesenchyme	Fin ray mesenchyme (not in all fin rays)
***Tg(2P****Δ****epi-EIand1*:*EGFP)***	mesenchyme	Fin ray mesenchyme (bright and in all fin rays)
**1**^**st**^ **Exon + Intron (EI)**	Adult tissue transcriptional enhancer + alternative promoter	

As development progresses to adulthood, reporter expression in the *Tg(2Pand1*:*eGFP)* and *Tg(epi*.*and1-βG*:*eGFP)* lines disappeared, which was problematic for the analysis of *and1* expression during adult fin regeneration. The inclusion of *EI* in *Tg(2P-EIand1*:*eGFP)*, *Tg((1117–1)EIand1*:*eGFP)* and *Tg(2PΔepi-EIand1*:*eGFP)* lines was found to significantly boost tissue-specific expression in adult zebrafish, suggesting that adequate *and1* expression relies on a potential enhancer situated within the *EI* region. The inclusion of *EI* allowed us to successfully generate a transgenic line, *Tg((1117–1)EIand1*:*eGFP)*, that specifically reports *and1* expression within the basal epithelial layer during adult fin regeneration. In addition to this observed boost in *and1* reporter lines, the *EI* region can be paired with a weak enhancer, such as the *shha aRC* enhancer, to drive reporter expression within the floor plate cells of the neural tube suggesting the presence of an alternative promoter in *EI*. Therefore, the inclusion of *EI* not only allowed us to generate *and1* reporter lines that can be used for adulthood analyses, but also to generate reporter lines using other enhancers. There are several findings that support the notion of intron-mediated enhancement. For example, the inclusion of an intron of certain genes in reporter constructs significantly increased transgene expression levels in mice, *Drosophila*, plants and zebrafish [28–31]. It was suggested that the inclusion of the first intron in reporter constructs can individually enhance each step of the dogma from transcription to translation and inhibit mechanisms that suppress these steps [32–34]. Intronic sequences may contain various binding sites for transcription factors that are highly expressed in many tissues [35]. Introns may also allow for interactions to occur between the TFIIH factor of the initiation complex and the U1 snRNA to prompt the re-initiation of transcription [36]. Other studies proposed that the inclusion of an intron in reporter constructs may oppose epigenetic silencing to which some foreign DNA elements in zebrafish are susceptible [31, 37, 38]. There are also studies that support the possibility of *EI* containing an alternative promoter that is necessary for driving sufficient *and1* expression in adults. Previously, it has been shown that alternative promoters can play an important role in boosting mRNA expression levels and/or conferring increased mRNA stability. Alternative promoters were, additionally, shown to increase translational efficiency and provide differential tissue-specific expression in yeast, zebrafish and humans [39–43]. Altogether, evidence suggests the *EI* region contains a strong transcriptional enhancer that may be comprised of various intronic enhancers and an alternative promoter, all of which render *EI* a useful molecular tool for boosting transgene expression in adulthood.

### Candidate transcription factors associated with the *epi3* element and *EI* region in regulating the dynamics of actinotrichia during regeneration

The *epi* region was discovered to likely contain a repressor that functions in suppressing mesenchymal-specific expression during the early stages of regeneration. Among *Tg(2P-EIand1*:*eGFP)*, *Tg(2Pand1*:*eGFP)*, *Tg((1117–1)EIand1*:*eGFP)*, *Tg(2PΔepi*.*and1*:*eGFP)*, and *Tg(2PΔepi3*.*and1*:*eGFP)*, the last two of the five exhibited strong reporter expression within the fin ray mesenchymal tissue between 2dpa and 4dpa. It was only after 4dpa, when fin ray mesenchymal-specific reporter expression was comparably bright to what was observed in the interray tissue in *Tg(2P-EIand1*:*eGFP)*, *Tg((1117–1)EIand1*:*eGFP)* and *Tg(2Pand1*:*eGFP)*. In 2dpa fin regenerates, actinotrichia are first seen in the regenerative tissue located above the interray tissue of the stump before it is formed in mass bundles along the regenerate [11]; this correlates with the emergence of *Tg(2P-EIand1*:*eGFP)* reporter expression in the interrays before fin ray-specific expression. This delay in actinotrichia formation in the regenerative fin ray tissue during the early stages of regeneration may be mediated by the *epi3* repressor sequence. In addition to this repressor sequence, *epi3* may also contain a basal epithelial-specific enhancer similar to the ectodermal enhancer that functions during embryonic and early larval development [14]. The *epi3* site contains putative binding sites for the TCF proteins, which have recently been shown to possess dual enhancing and repressing functions [44]. It was noted that TCF-4 as well as other TCF proteins, such as TCF3a and TCF3b, seem to have complementary expression patterns to mesenchymal-specific *Tg(2P-EIand1*:*eGFP)* and basal epithelial-specific *Tg((1117–1)EIand1*:*eGFP)* reporter expression throughout regeneration [45]. This complementary expression pattern suggests that these transcription factors may act on *epi3* in order to inhibit *and1* expression in specific regions of the outgrowing regenerate. Potential factors that may enhance basal epithelial-specific *and1* expression include TCF1 and LEF1, both of which also belong to the TCF/LEF family and are strongly expressed in the basal epithelial layer in the distal and distal-most regions of the regenerate [45, 46]. This overlap in expression with *Tg((1117–1)EIand1*:*eGFP)* reporter expression, suggest that TCF1 and LEF1 may enhance basal epithelial-specific *and1* expression throughout regeneration [45].

TCF proteins are generally downstream effectors of Wnt/β-catenin signaling [45, 46]. The activity of this pathway in *and1*-expressing cells is believed to directly and indirectly mediate cell proliferation within the blastema and osteoblast differentiation [45, 47, 48]. However, because of the various indirect functions of Wnt/β-catenin signaling [45], it is not certain whether or not the potential repressor effects of TCF proteins on *and1* expression can be associated with a negative effect of Wnt/β-catenin signaling on *and1* expression.

The *EI* region is believed to contain various intronic enhancers that may be necessary for adequate *and1* expression throughout adulthood and fin regeneration. TRANSFAC analysis revealed putative binding sites for OCT4, DLX5, HOX, MSXE and LEF1 proteins within the *EI* region. Previous work on the genes encoding these proteins suggest they are plausible candidates for *actinodin1* adult and regeneration-specific activation [43, 49–52]. The expression pattern of *oct4* in zebrafish fin regeneration has yet to be characterized; however, stemming from the fact that this gene is documented to have an indispensable role in regulating cell pluripotency in stem cells, it is possible that *oct4* may be reactivated in regenerative tissues that express *and1* [49, 53]. The *dlx5* genes include *dlx5a* which is expressed within the basal wound epidermis and likely overlaps with the *and1*-expressing cells within the basal epithelial layer that lines the distal-most regions of the regenerate [50]; therefore, *dlx5a* is a likely candidate for an activator of *and1* in the basal epithelial layer. The *msxe* gene belongs to the *msx* homeobox gene family, which were found to be highly expressed in the blastema, hence the likelihood of *msxe* being a candidate for an activator of *and1* in the mesenchyme [51]. As previously described, *lef1* is expressed in the basal epithelial layer within the distal-most region of the regenerate and may either be an activator of basal epithelial layer-specific *and1* expression via the *epi3* site or via the *EI* region [45]. Finally, the 5’*hoxA/D* genes are heavily involved in proximal-distal patterning in fin/limb development [54, 55] and we have previously provided evidence that *actinodin1* may in fact be regulated by one or more 5’HoxA/D proteins in pectoral and median in fold mesenchyme [56]. Furthermore, the *Hox* genes are expressed in regenerative tissues in planarians, *Xenopus*, urodele amphibians and zebrafish [57–60]. Unfortunately, the expression patterns of most *hox* genes have yet to be characterized in the zebrafish fin regenerate. As of now, it has been shown that *hoxa13a*, *hoxa13b*, *hoxc13a* and *hoxc13b* genes are highly upregulated in the blastema, basal epithelial layer and/or osteoblasts in the regenerate [52, 60, 21], suggesting that *hox* genes may also be potential activators of *and1* expression.

## Conclusion

While actinotrichia are absent in mammals, they play a crucial role in cell migration, cell differentiation and structural support in zebrafish during fin development and regeneration. Therefore, understanding the dynamics and distribution of actinotrichia-forming cells is indispensable to the study of zebrafish fin regeneration. In the present study, we have characterized additional *cis*-acting regulatory elements of *actinodin1* that are active during adult fin regeneration. The identification of these regulatory elements has allowed us to generate a transgenic line that fully recapitulates *actinodin1* expression and to further explore potential molecular pathways that may govern the dynamics of actinotrichia formation during adult fin regeneration. Finally, the discovery of an adult-specific, transcriptional enhancer has provided us with an additional molecular tool that may be useful for the conception of transgenic lines that can be studied during adulthood.

## Supporting information

S1 Fig *Tg(2P-EIand1*:*eGFP)* recapitulates endogenous *and1* expression in developing caudal fin at juvenile stage. All experiments were performed on transverse cryosections of middle region (A-A’, B-B’, E, G) and distal region (C, D, F, H) of developing caudal fin of 40dpf juvenile fish. *In situ* hybridization (n = 8) for *eGFP* of *Tg(2P-EIand1*:*eGFP)* (A-A’, C, C’) and endogenous *and1* expression (B-B’, D, D’). The fin rays and the interrays are indicated by brackets. (A’-D’) Higher magnification of a single fin ray from panels A-D, respectively. (E, F) Immunohistochemistry (n = 9) for And1/2. (E’, F’) merge with DAPI staining, which stains cell nuclei. n = # of fish of which fins were sectioned, and on which given experiment was performed. e: epithelium; fr: fin ray; ir: interray; m: mesenchyme. Melanocytes are the dark spots indicated by an asterisk. Scale bars: Panel A, B, C, D = 20μm, A’, B’, C’, D’ = 10μm, E-E’, F-F’ = 20μm.
(TIF)

S2 Fig Time course analysis of eGFP in *Tg(epi*.*and1-βG*:*eGFP)* throughout regeneration. Regenerative time course analysis of *in vivo* reporter expression of *Tg(epi*.*and1-βG*:*eGFP)* (n = 25 fish/line, 2 lines) at 2dpa (A), 3dpa (B), 4dpa (C), 7dpa (D), 16-17dpa (E) and 24-25dpa (F). (A-F) There is a complete absence of reporter expression throughout regeneration. All scale bars = 200μm.
(TIF)

S3 Fig Time course analysis of *Tg((1117–1)EIand1*:*eGFP)* and *Tg(2P-EIand1*:*eGFP)* throughout regeneration. *In vivo* time course analysis of reporter expression of *Tg(2P-EIand1*:*eGFP)* (n = 12) (A-F) and *Tg((1117–1)EIand1*:*eGFP)* (n = 5) (n = 25 fish/line, 2 lines) (G-L) during fin regeneration. (A-B, G-H) At 2dpa and 3dpa, reporter expression is brightly observed only in interray tissue of *Tg(2P-EIand1*:*eGFP)* and *Tg((1117–1)EIand1*:*eGFP)*. (C-D) At 4dpa and 7dpa, fin ray mesenchymal-specific reporter expression occurs along the proximal-distal axis and is as equally bright as that of interray-specific expression in *Tg(2P-EIand1*:*eGFP)*, which occurs at the distal region of the regenerate. (E-F) Fin ray-specific reporter expression begins to distally restrict in *Tg(2P-EIand1*:*eGFP)*. (I-J) Reporter expression only occurs in interray of *Tg((1117–1)EIand1*:*eGFP)* and always remains confined within the distal region of regenerate. (K-L) reporter expression occurs in the interray and distal edge of the fin in *Tg((1117–1)EIand1*:*eGFP)*. Scale bar: A, G = 100μm, B, H = 200μm, C, I = 200μm, D, J = 200μm, E-F, K-L = 200μm. (Images in panels A-F have been reused from Fig 3.4. for better comparison of eGFP expression.)
(TIF)

S4 Fig Transverse sections of 4dpa of *Tg(1117–1+EI*: *eGFP)*. Reporter expression of (A) *Tg((1117–1)EIand1*:*eGFP)* (n = 2) *in vivo* at 4dpa. Immunostaining for eGFP in *Tg((1117–1)EIand1*:*eGFP)* on consecutive transverse cryosections (B-F). (B) In the most distal region of regenerate, reporter expression occurs in the basal epithelial layer distal to the hemirays. As sections progress proximally, (C-F) reporter expression within basal epithelial layer restricts towards interray region. (E, F) Reporter expression occurs within basal epithelial layer of the interray and the fin ray region closer to the interray (indicated by dotted yellow box). It is absent in middle regions of hemirays. Basal epithelial layer-specific expression is indicated by yellow arrows. Pink star indicates autofluorescence from blood vessels. Scale bars: A = 200μm, B-F = 50μm. n = # of fish from which sections were obtained.
(TIF)

S5 Fig The *EI* region is necessary to enhance fin ray mesenchymal-specific expression even without the epi repressor. (A) Distal fin ray mesenchymal-specific expression does not occur in all fin rays. Reporter expression is bright and consistent in all fin rays in *Tg(2P-EIand1*:*eGFP)* (B), and in two different *Tg(2PΔepi-EIand1*:*eGFP)* lines (C, D). Red arrows indicate presence of fin ray-specific expression while asterisk indicates absence of expression. Scale bar = 200μm.
(TIF)
