# Supplementary figures and images for: Differential actinodin1 regulation in embryonic development and adult fin regeneration in Danio rerio
Source: PLoS One. 2019 May 2;14(5):e0216370. doi: 10.1371/journal.pone.0216370 (PMC6497306; doi:10.1371/journal.pone.0216370)

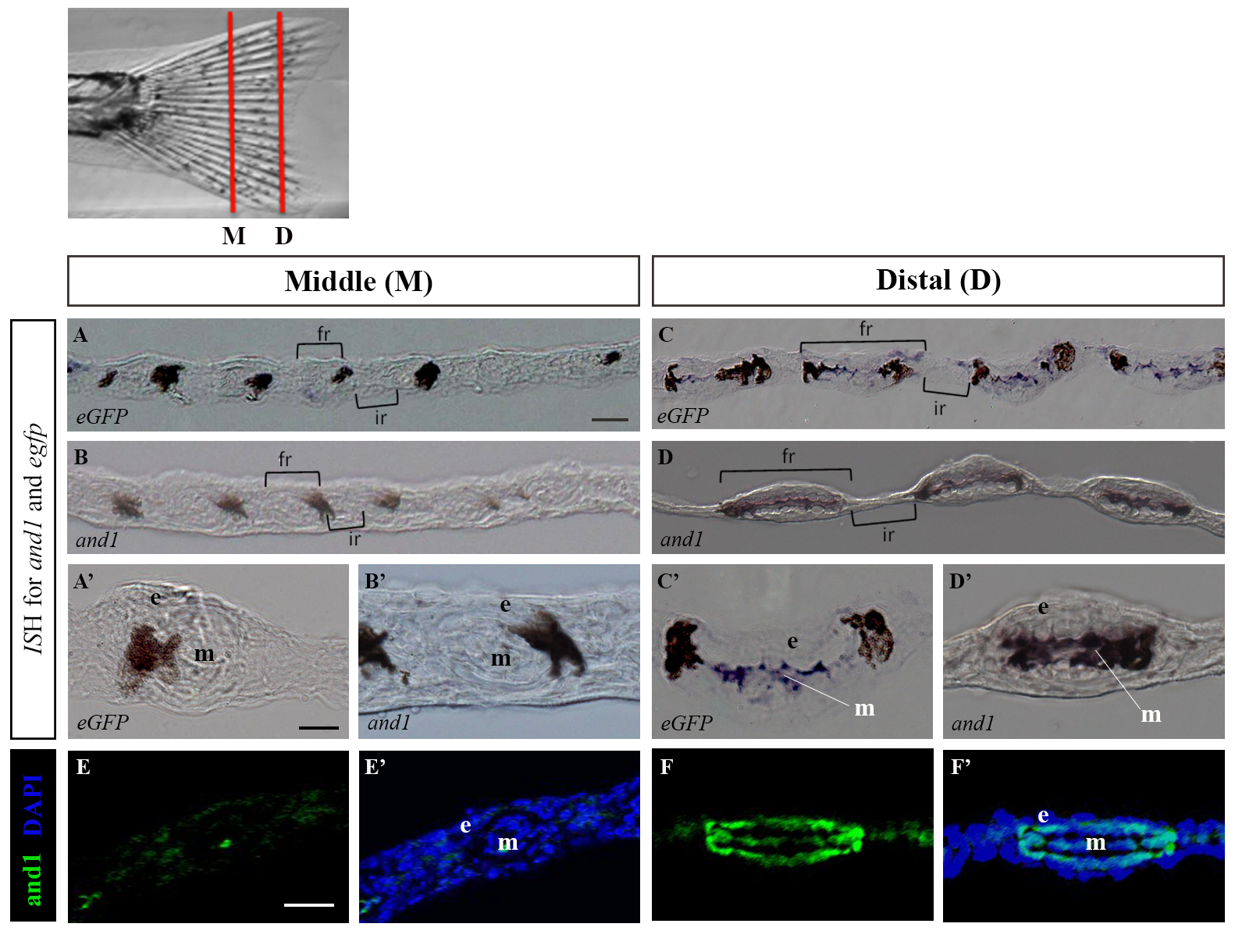

Supplement: S1 Fig — All experiments were performed on transverse cryosections of middle region (A-A’, B-B’, E, G) and distal region (C, D, F, H) of developing caudal fin of 40dpf juvenile fish. In situ hybridization (n = 8) for eGFP of Tg(2P-EIand1:eGFP) (A-A’, C, C’) and endogenous and1 expression (B-B’, D, D’). The fin rays and the interrays are indicated by brackets. (A’-D’) Higher magnification of a single fin ray from panels A-D, respectively. (E, F) Immunohistochemistry (n = 9) for And1/2. (E’, F’) merge with DAPI staining, which stains cell nuclei. n = # of fish of which fins were sectioned, and on which given experiment was performed. e: epithelium; fr: fin ray; ir: interray; m: mesenchyme. Melanocytes are the dark spots indicated by an asterisk. Scale bars: Panel A, B, C, D = 20μm, A’, B’, C’, D’ = 10μm, E-E’, F-F’ = 20μm. (TIF) [file pone.0216370.s001.tif]

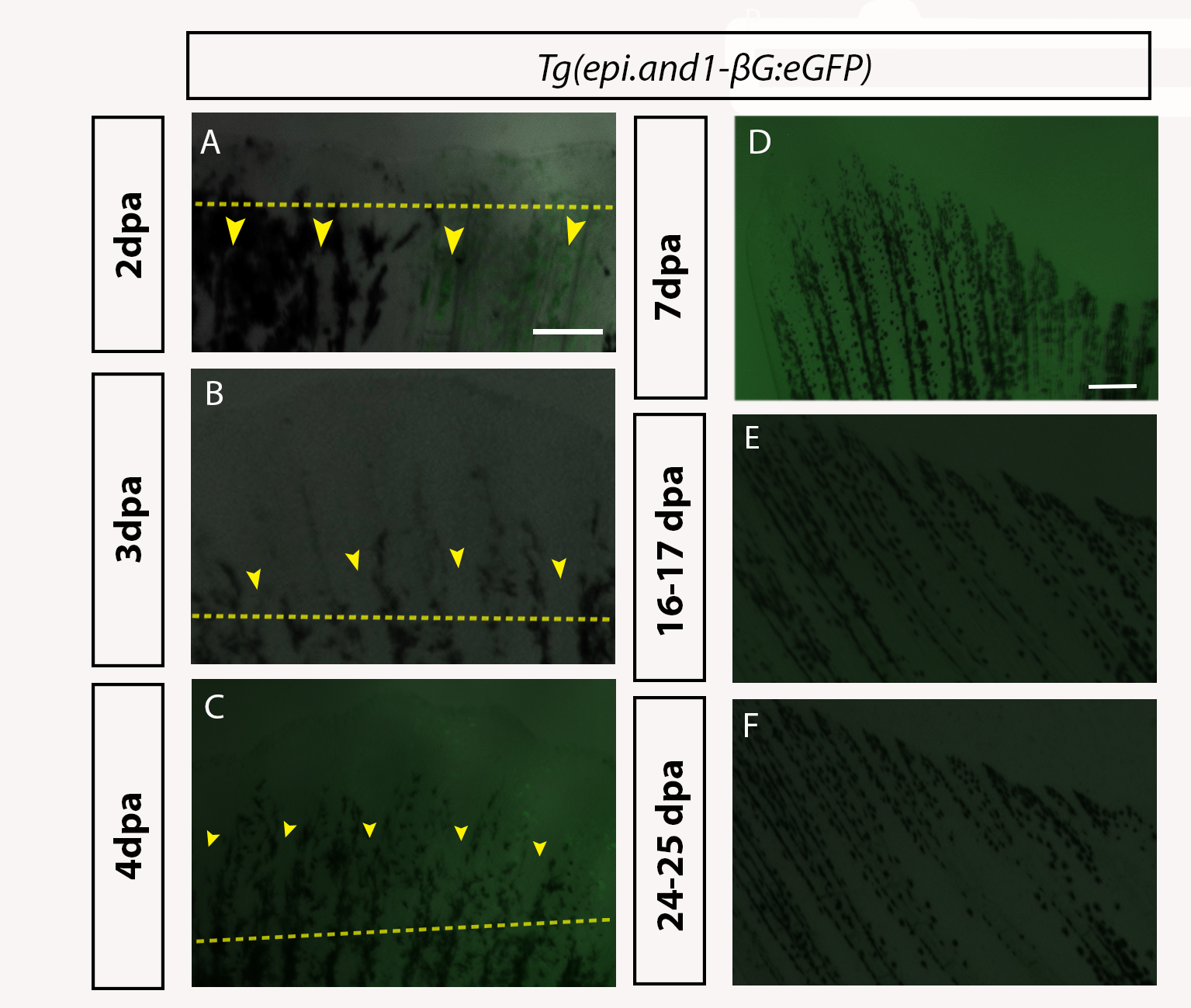

Supplement: S2 Fig — Regenerative time course analysis of in vivo reporter expression of Tg(epi.and1-βG:eGFP) (n = 25 fish/line, 2 lines) at 2dpa (A), 3dpa (B), 4dpa (C), 7dpa (D), 16-17dpa (E) and 24-25dpa (F). (A-F) There is a complete absence of reporter expression throughout regeneration. All scale bars = 200μm. (TIF) [file pone.0216370.s002.tif]

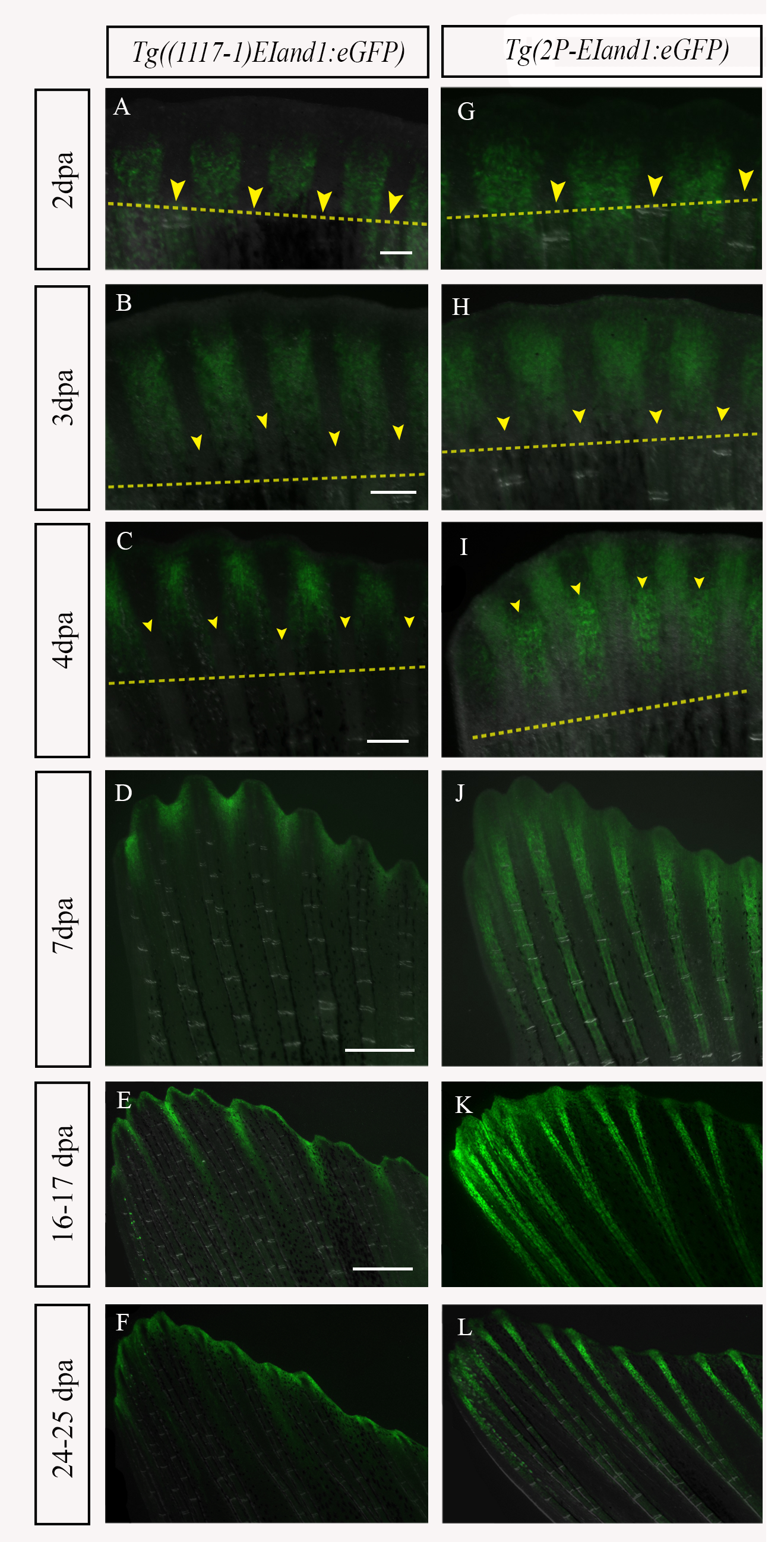

Supplement: S3 Fig — In vivo time course analysis of reporter expression of Tg(2P-EIand1:eGFP) (n = 12) (A-F) and Tg((1117–1)EIand1:eGFP) (n = 5) (n = 25 fish/line, 2 lines) (G-L) during fin regeneration. (A-B, G-H) At 2dpa and 3dpa, reporter expression is brightly observed only in interray tissue of Tg(2P-EIand1:eGFP) and Tg((1117–1)EIand1:eGFP). (C-D) At 4dpa and 7dpa, fin ray mesenchymal-specific reporter expression occurs along the proximal-distal axis and is as equally bright as that of interray-specific expression in Tg(2P-EIand1:eGFP), which occurs at the distal region of the regenerate. (E-F) Fin ray-specific reporter expression begins to distally restrict in Tg(2P-EIand1:eGFP). (I-J) Reporter expression only occurs in interray of Tg((1117–1)EIand1:eGFP) and always remains confined within the distal region of regenerate. (K-L) reporter expression occurs in the interray and distal edge of the fin in Tg((1117–1)EIand1:eGFP). Scale bar: A, G = 100μm, B, H = 200μm, C, I = 200μm, D, J = 200μm, E-F, K-L = 200μm. (Images in panels A-F have been reused from Fig 3.4. for better comparison of eGFP expression.) (TIF) [file pone.0216370.s003.tif]

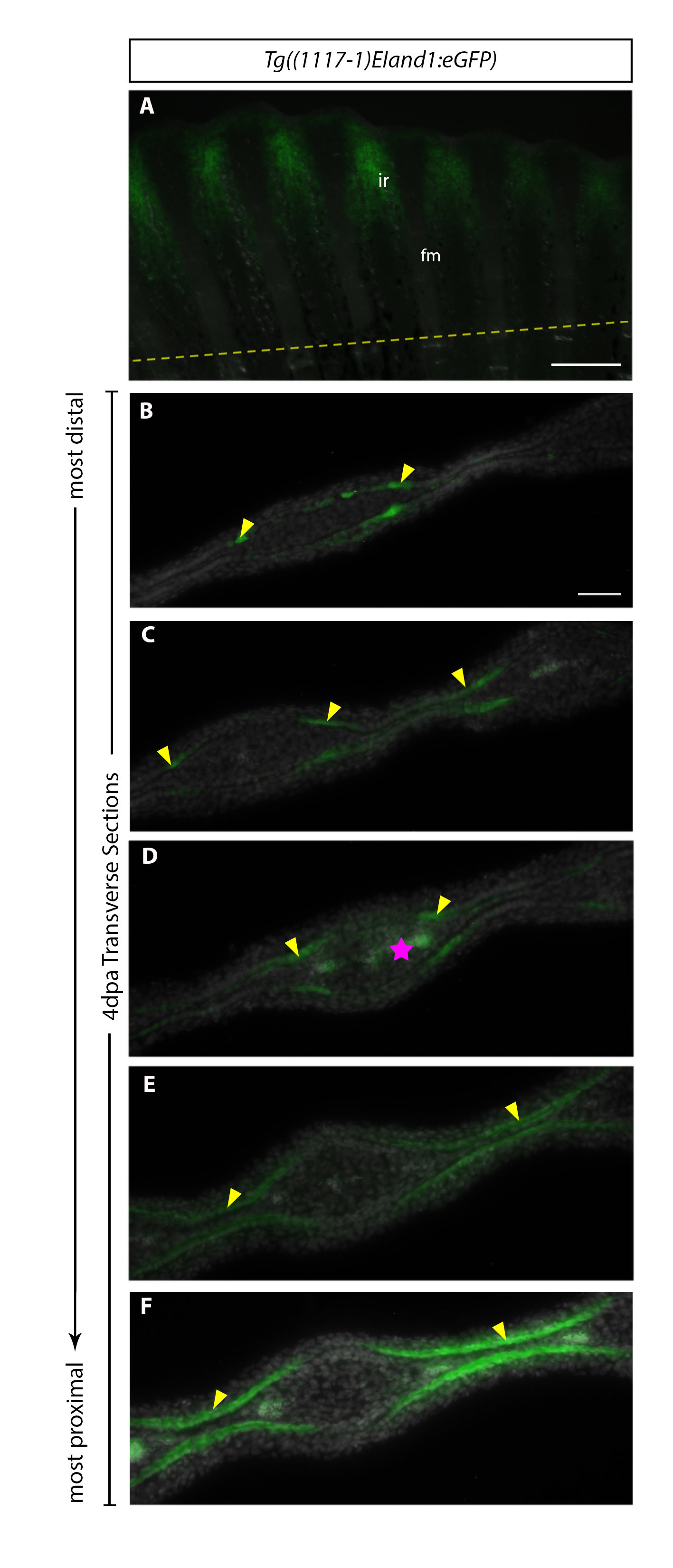

Supplement: S4 Fig — Reporter expression of (A) Tg((1117–1)EIand1:eGFP) (n = 2) in vivo at 4dpa. Immunostaining for eGFP in Tg((1117–1)EIand1:eGFP) on consecutive transverse cryosections (B-F). (B) In the most distal region of regenerate, reporter expression occurs in the basal epithelial layer distal to the hemirays. As sections progress proximally, (C-F) reporter expression within basal epithelial layer restricts towards interray region. (E, F) Reporter expression occurs within basal epithelial layer of the interray and the fin ray region closer to the interray (indicated by dotted yellow box). It is absent in middle regions of hemirays. Basal epithelial layer-specific expression is indicated by yellow arrows. Pink star indicates autofluorescence from blood vessels. Scale bars: A = 200μm, B-F = 50μm. n = # of fish from which sections were obtained. (TIF) [file pone.0216370.s004.tif]

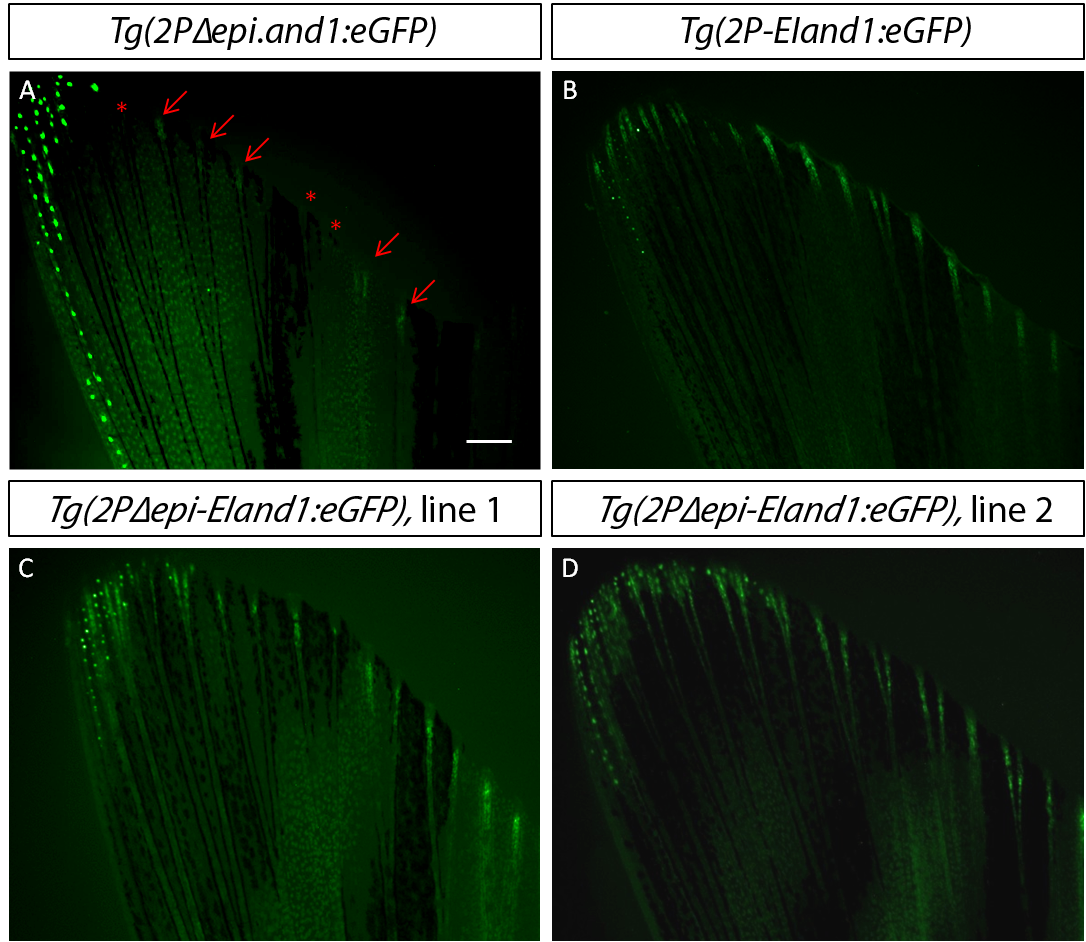

Supplement: S5 Fig — (A) Distal fin ray mesenchymal-specific expression does not occur in all fin rays. Reporter expression is bright and consistent in all fin rays in Tg(2P-EIand1:eGFP) (B), and in two different Tg(2PΔepi-EIand1:eGFP) lines (C, D). Red arrows indicate presence of fin ray-specific expression while asterisk indicates absence of expression. Scale bar = 200μm. (TIF) [file pone.0216370.s005.tif]
